# Elevated TRIM25 Impairs Poly (ADP‐ribose) Metabolism via PARG Degradation and Mediates Compression‐Induced Intervertebral Disc Degeneration

**DOI:** 10.1002/advs.202519248

**Published:** 2026-02-17

**Authors:** Zhangrong Cheng, Haiyang Gao, Wenbo Wu, Pengzhi Shi, Xianglong Chen, Zimu Yu, Wang Wu, Kangcheng Zhao, Cao Yang, Yukun Zhang

**Affiliations:** ^1^ Department of Orthopedics Union Hospital Tongji Medical College Huazhong University of Science and Technology Wuhan Hubei China

**Keywords:** intervertebral disc degeneration, low back pain, nucleus pulposus cells

## Abstract

Intervertebral disc degeneration (IVDD) is a leading global cause of low back pain, with abnormal mechanical stress being a central contributing factor. However, the molecular mechanisms through which mechanical stress signals are transduced into intracellular pathological responses to drive degeneration remain poorly understood. This study demonstrates that mechanical compression induces DNA damage and triggers parthanatos—a caspase‐independent form of cell death driven by toxic accumulation of poly(ADP‐ribose) (PAR) polymers. Mechanistically, mechanical compression upregulates the E3 ubiquitin ligase TRIM25, which directly binds to and promotes the ubiquitination and degradation of poly(ADP‐ribose) glycohydrolase (PARG), leading to disrupted PAR metabolism and toxic PAR accumulation. Concurrently, TRIM25 targets the DNA repair protein Ku80 for degradation, exacerbating genomic instability and activating the RIG‐I innate immune pathway, thereby inducing the release of inflammatory factors. Thus, under mechanical stress, TRIM25 acts as a key node coordinating DNA damage, cell death, and inflammatory responses, forming a multi‐mechanistic network that promotes IVDD progression. In a rat model of compression‐induced IVDD, restoring PAR homeostasis by targeting the TRIM25–PARG axis significantly attenuated disc degeneration, suggesting the therapeutic potential of targeting this pathway in IVDD.

## Graphical abstract

TRIM25 acts as a multifunctional hub driving intervertebral disc degeneration under mechanical stress. Mechanical compression significantly upregulates TRIM25 expression, establishing it as a key E3 ubiquitin ligase platform. TRIM25 targets PARG and Ku80 via distinct molecular interfaces, triggering their ubiquitination and degradation. This process synergistically promotes genomic instability and dysregulates PAR metabolism, thereby accelerating parthanatos in NPCs and the progression of IVDD.

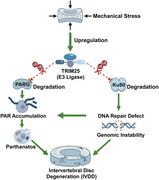

## 1 Introduction

Intervertebral disc degeneration (IVDD) is the major pathological cause of disability and chronic low back pain worldwide [1]. Its typical pathological features include a gradual decrease in the number of nucleus pulposus cells (NPCs), a persistent low‐grade inflammatory response, and a severe imbalance in the synthesis and degradation of the extracellular matrix [2]. Among the multiple pathogenic factors of IVDD, abnormal biomechanics, especially chronic or acute excessive mechanical stress, is considered to be the core physical trigger for initiating and accelerating the process of IVDD [3]. Regulated cell death plays a key role in the progression of IVDD, involving a highly organized signaling cascade and specific molecular mechanisms. Early studies focused on mechanical stress‐induced apoptosis in NPCs through mitochondrial or death receptor pathways [4, 5]. However, recent studies have shown that under high‐intensity or sustained mechanical stress, NPCs exhibit significant diversity in their death pathways, including PANoptosis [6, 7] and ferroptosis [8, 9]. Furthermore, mechanical stress can induce DNA damage in NPCs, establishing a positive feedback loop with the local inflammatory microenvironment, forming a complex molecular network that synergistically drives the progression of IVDD [10, 11]. Therefore, further elucidating the upstream molecular regulatory mechanisms by which mechanical stress coordinates these multiple pathological phenotypes is crucial for a comprehensive understanding of the pathogenesis of IVDD and the development of targeted and effective therapeutic strategies.

Poly(ADP‐ribose) (PAR) synthesis is initiated when DNA damage activates enzymes of the PARP family, primarily PARP1. This enzyme utilizes nicotinamide adenine dinucleotide (NAD^+^) as a substrate to catalyze the synthesis of PAR chains, which are covalently attached to itself or target proteins—a process termed PARylation [12]. Under physiological conditions, PARylation facilitates the recruitment of DNA repair proteins, thereby maintaining genomic stability. However, under pathological conditions, dysregulated PAR metabolism leads to abnormal accumulation of PAR, which can trigger a caspase‐independent form of regulatory cell death known as parthanatos [13, 14]. The role of parthanatos in neurodegenerative diseases and ischemia‐reperfusion injury has been extensively studied [15, 16]. Its molecular initiation mechanism often stems from a functional imbalance between hyperactivation of PARP—induced by severe DNA damage—and its negative regulator, poly(ADP‐ribose) glycohydrolase (PARG) [17]. As the key enzyme responsible for degrading PAR polymers and maintaining PAR metabolic homeostasis, loss of PARG activity directly results in toxic accumulation of PAR, leading to cellular energy depletion and death [18]. Studies have shown that aberrant PAR accumulation mediates cytotoxicity through multiple mechanisms: (1) it inhibits NAD^+^ regeneration pathways, persistently depleting intracellular NAD^+^ reserves and ultimately causing a severe energy crisis [13]; (2) it induces the release of apoptosis‐inducing factor (AIF) from mitochondria, thereby driving macrophage migration inhibitory factor (MIF)‐mediated DNA cleavage and contributing to parthanatos [19]; and (3) high concentrations of PAR can further impede normal DNA damage repair processes by interfering with various nucleic acid and protein functions [20]. Although studies have reported the presence of DNA damage and PARP activation in degenerated intervertebral disc tissues [21, 22], the regulatory mechanisms and functional implications of PAR expression in IVDD, particularly under mechanical stress, remain unclear. On the other hand, NPCs under mechanical stress not only exhibit a significant propensity for cell death but are also frequently accompanied by reduced DNA repair capacity and a robust inflammatory response. However, it remains unknown whether these pathological phenomena represent independent parallel events or are orchestrated by common upstream regulatory molecules.

Based on this background, this study aims to systematically investigate the integrated molecular mechanisms underlying mechanical stress—specifically compressive stress (hereinafter referred to as compression or COMP)‐induced death of NPCs and dysfunction. Our transcriptome sequencing analysis revealed that compressive stress induces significant DNA damage and cell death in NPCs. This form of cell death aligns with the key characteristics of parthanatos, manifesting as caspase independence and pronounced accumulation of PAR polymers. Further investigation indicated that this PAR accumulation stems from the specific post‐translational downregulation of PARG. To elucidate the mechanism governing PARG instability, we employed a proteomic approach using co‐immunoprecipitation (Co‐IP) coupled with mass spectrometry (MS), identifying TRIM25 as a prominent interacting partner of PARG. Crucially, this discovery was corroborated by our transcriptomic profiling, which showed significant upregulation of TRIM25 in NPCs under mechanical stress. This convergence of proteomic interaction data and pathological expression patterns provided a compelling rationale to identify TRIM25 as a candidate upstream regulator. Previous research on TRIM25 has primarily focused on its role in antiviral innate immune responses, particularly its regulation of the RIG‐I signaling pathway [23]. In the context of IVDD, recent studies have begun to uncover the involvement of other TRIM family members [24]. For instance, Deng et al. reported that TRIM21 drives intervertebral disc degeneration induced by oxidative stress via mediating HIF‐1α degradation [25]. Similarly, TRIM32 has been identified as a regulator of inflammatory apoptosis; it promotes nucleus pulposus cell death in response to TNF and IL1B stimulation by ubiquitinating AXIN1 to trigger β‐catenin signaling [26]. However, these studies have predominantly focused on biochemical triggers (e.g., oxidative stress, inflammatory cytokines) and classical cell death modes such as apoptosis. Whether TRIM proteins can function as mechanotransducers to directly link mechanical stress to non‐canonical death pathways, specifically parthanatos, remains largely unexplored. Distinct from previous findings, this study demonstrates that COMP‐induced TRIM25 upregulation not only mediates the ubiquitin‐dependent degradation of PARG but also impairs DNA damage repair by facilitating Ku80 degradation, while simultaneously promoting inflammation via the RIG‐I pathway. These findings suggest that TRIM25 functions as a central hub, coordinately regulating DNA damage, cell death, and inflammation within the mechanical stress microenvironment. To our knowledge, this study is the first to reveal the ubiquitination‐mediated regulation of PARG stability and to identify TRIM25 upregulation as a central mechanism driving multiple pathological phenotypes in IVDD under mechanical stress.

## 2 Results

### 2.1 Mechanical Compression Triggers a Multimodal Cell Death Program Involving PANoptosis and DNA Damage‐Driven Defects

**FIGURE 1:**
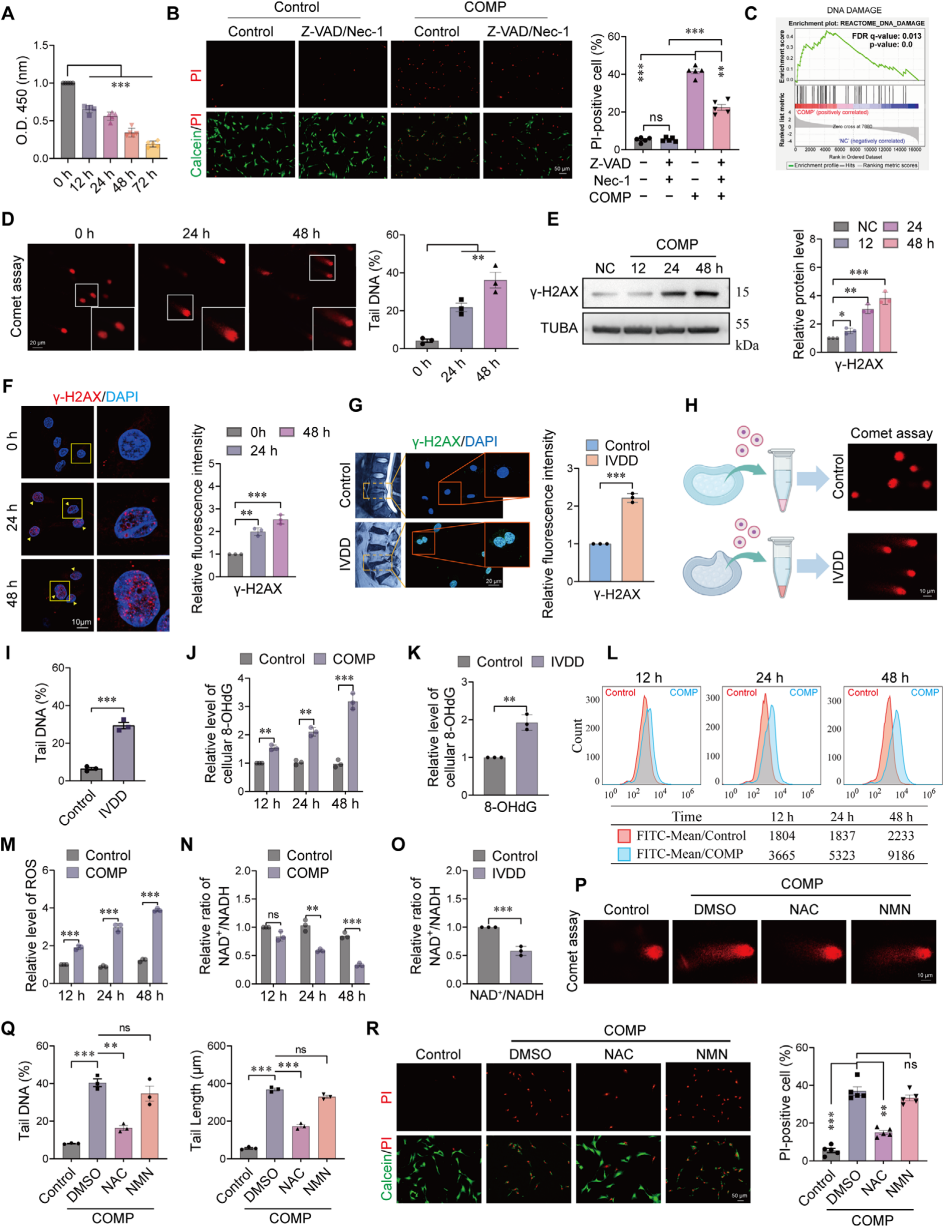
Mechanical compression triggers a multimodal cell death program involving PANoptosis and DNA damage‐driven defects. (A) Viability of NPCs subjected to mechanical compression at different time points (12, 24, 48, 72 h), as assessed by CCK‐8 assay. n = 5. (B) Calcein‐AM/PI staining showing the effect of the combined treatment of Z‐VAD (20 µm) and Nec‐1 (20 µm) on compression‐induced cell death. Scale bar: 50 µm. n = 5. (C) Gene Set Enrichment Analysis (GSEA) of RNA‐seq data from compressed NPCs, showing significant activation of the DNA damage response pathway. (D) Representative images (left) and quantitative analysis (right) of comet assays indicating increased DNA damage in NPCs after 24 and 48 h of compression. Scale bar: 20 µm. n = 3. (E–F) Western blot (E) and immunofluorescence staining (F) showing upregulation of the DNA damage marker γ‐H2AX in compressed NPCs. Scale bar: 10 µm. n = 3. (G) Immunofluorescence of human intervertebral disc sections revealing a positive correlation between γ‐H2AX signal intensity and the degree of disc degeneration. Scale bar: 20 µm. n = 3. (H–I) Comet assays demonstrating higher baseline DNA damage in primary NPCs isolated from degenerated compared to healthy discs. Scale bar: 10 µm. n = 5. (J) ELISA showing elevated 8‐OHdG levels in compressed NPCs, indicative of oxidative DNA damage. n = 3. (K) Significantly higher levels of 8‐OHdG in human degenerated nucleus pulposus tissues compared to healthy controls. n = 3. (L–M) Representative flow cytometry plots (L) and quantitative analysis (M) showing a compression‐induced increase in intracellular ROS levels. n = 3. (N) NAD^+^/NADH ratio in NPCs decreased after 24 and 48 h of compression. n = 3. (O) The NAD^+^/NADH ratio was significantly lower in human degenerated disc tissues than in healthy controls. n = 3. (P–Q) Comet assays showing that the ROS scavenger NAC (1 mm), but not the NAD^+^ precursor NMN (0.5 mm), attenuated compression‐induced DNA damage. Scale bar: 10 µm. n = 3. (R) Calcein‐AM/PI staining evaluating the effects of NAC and NMN on NPC death under compression. Scale bar: 50 µm. n = 5. Data are presented as mean ± SEM. ^*^ *p* < 0.05, ^**^ *p* < 0.01, ^***^ *p* < 0.001, and ns means not significant.

To investigate the impact of mechanical stress on the survival of NPCs, we first applied 1.0 MPa of static compression to human NPCs cultured in vitro [27]. A time‐dependent significant decrease in cell viability was observed, reaching its lowest level after 72 h of treatment (Figure 1A). To characterize the nature of cell death, we initially employed the pan‐caspase inhibitor Z‐VAD. However, Calcein‐AM/PI staining results demonstrated that Z‐VAD pretreatment failed to effectively prevent compression‐induced NPC death (Figure S1A). To further characterize the nature of this cell death, we examined specific molecular markers. Western blot analysis revealed that mechanical stress significantly upregulated cleaved caspase‐3, the N‐terminal fragment of GSDMD (GSDMD‐N, a marker of pyroptosis), and phosphorylated MLKL (p‐MLKL, a marker of necroptosis) (Figure S1B,C). Notably, while Z‐VAD treatment inhibited caspase‐3 and GSDMD‐N, it failed to reduce p‐MLKL protein levels (Figure S1B,C). These findings indicate the activation of PANoptosis, a coordinated cell death program, and explain the limited efficacy of Z‐VAD alone. To determine if PANoptosis was the sole driver of cell death, we treated cells with a combination of Z‐VAD and the RIPK1 inhibitor Necrostatin‐1 (Nec‐1). As shown in Figure 1B, although this combination effectively blocked the key pathways of PANoptosis, a substantial fraction of cells still underwent cell death, and viability remained significantly lower than in controls. The persistence of cell death despite the blockade of PANoptosis pathways suggested that an alternative, caspase‐ and RIPK‐independent mechanism plays a critical role. To elucidate the specific underlying mechanism, we performed transcriptome sequencing on compressed cells. Gene Set Enrichment Analysis (GSEA) revealed a significant enrichment of gene sets related to the DNA damage response (Figure 1C), implying that DNA damage might be the driver of this residual, drug‐resistant cell death. We directly confirmed this by comet assay, which showed a significant increase in the percentage of tail DNA in NPCs after 24 and 48 h of compression (Figure 1D). Western Blot and immunofluorescence staining further indicated that both the protein level and fluorescence intensity of γ‐H2AX, a key marker of DNA double‐strand breaks, were strongly induced (Figure 1E,F). We further validated the clinical relevance of these findings. Staining of human intervertebral disc (IVD) tissue sections revealed that the signal intensity of γ‐H2AX was positively correlated with the degree of disc degeneration (Figure 1G). Furthermore, primary NPCs isolated from degenerated discs also exhibited higher baseline levels of DNA damage (Figure 1H,I), supporting the pathological link between DNA damage and IVDD.

We further explored the source of DNA damage. Compression induced a sharp increase in intracellular reactive oxygen species (ROS) levels in human NPCs (Figure 1L,M) and led to a significant accumulation of the oxidative DNA damage marker 8‐hydroxy‐2'‐deoxyguanosine (8‐OHdG) both in cells (Figure 1J) and human degenerated disc tissues (Figure 1K), identifying oxidative stress as a primary driver of DNA damage. To determine the upstream origin of this oxidative stress, we examined mitochondrial integrity, given that mitochondrial dysfunction is a primary source of ROS production. As shown in Figure S1D, mechanical compression induced a significant, time‐dependent reduction in mitochondrial membrane potential (MMP), confirming that mitochondrial depolarization drives the observed ROS accumulation. Persistent DNA damage and PARP activation typically lead to depletion of intracellular NAD^+^. As expected, we found that compression significantly reduced the NAD^+^/NADH ratio in NPCs (Figure 1N), a phenomenon that was corroborated in degenerated human IVD tissues (Figure 1O). To establish the causal relationship among ROS, DNA damage, and cell death, rescue experiments were conducted. The comet assay showed that the ROS scavenger N‐acetylcysteine (NAC) effectively mitigated compression‐induced DNA damage, whereas supplementation with nicotinamide mononucleotide (NMN), a NAD^+^ precursor, had no such effect (Figure 1P,Q). Correspondingly, in cell viability assays, NAC treatment significantly reversed compression‐induced death of NPCs, while NMN provided no protective effect (Figure 1R). In summary, the results from this section demonstrate that mechanical compression triggers severe DNA damage and NAD^+^ depletion in NPCs by inducing oxidative stress, ultimately leading to a caspase‐independent cell death.

### 2.2 Abnormal Accumulation of PAR Mediates NPCs Parthanatos and Accelerates IVDD

**FIGURE 2:**
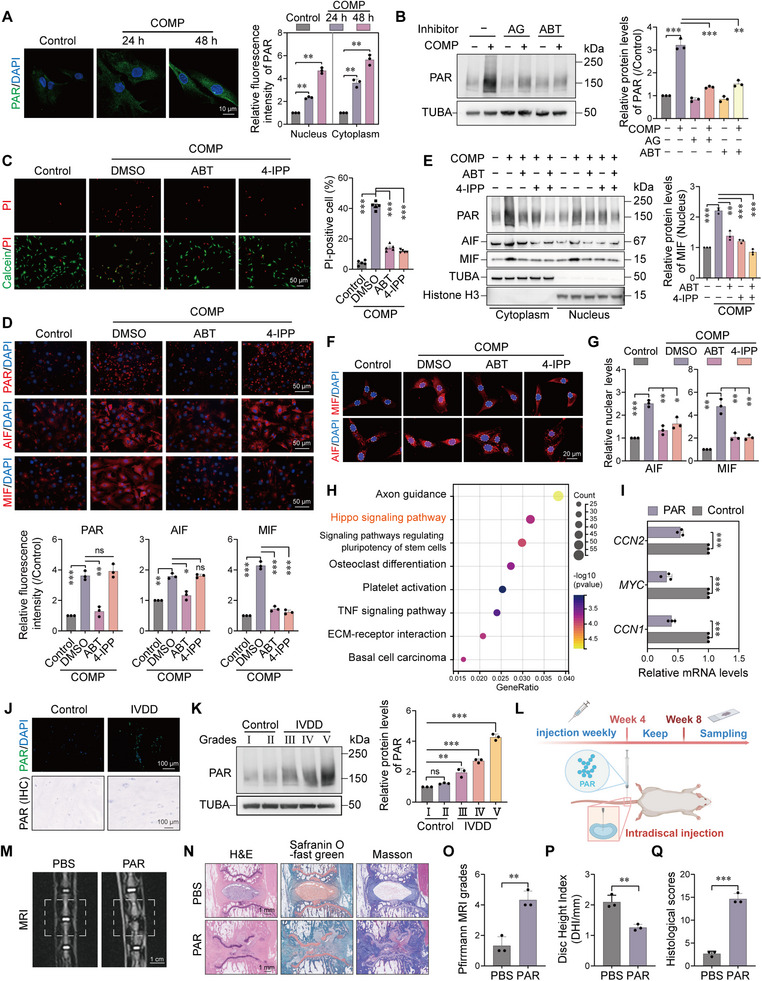
Abnormal accumulation of PAR mediates NPCs parthanatos and accelerates IVDD. (A) Immunofluorescence staining showing elevated PAR levels in NPCs after 24 and 48 h of mechanical compression. Scale bar: 10 µm. n = 3. (B) Western blot analysis demonstrating that pretreatment with PARP inhibitors AG (AG‐014699/Rucaparib, 1 µm) or ABT (ABT‐888/Veliparib, 10 µm) effectively reduced compression‐induced PAR accumulation. n = 3. (C) Viability of compressed NPCs treated with or without the PARP inhibitor ABT‐888, as assessed by Calcein‐AM/PI staining. Scale bar: 50 µm. n = 5. (D) Immunofluorescence staining showing the effects of ABT‐888 (10 µm) and the MIF inhibitor 4‐IPP (10 µm) on the levels of AIF, MIF, and PAR in compressed NPCs. Scale bar: 50 µm. n = 3. (E) Nuclear‐cytoplasmic fractionation assays indicated that ABT‐888 or 4‐IPP treatment prevented compression‐induced nuclear translocation of MIF. n = 3. (F‐G) Immunofluorescence staining showing the effects of ABT‐888 or 4‐IPP on the nuclear location of AIF and MIF. Scale bar: 20 µm. n = 3. (H–I) RNA sequencing and KEGG analysis revealed significant activation of the Hippo signaling pathway in NPCs treated with PAR polymer (10 µm) for 48 h (H). qPCR validation (I) showed downregulation of Hippo/Yap downstream target genes (*CCN2*, *MYC*, and *CCN1*). n = 3. (J–K) Immunofluorescence (J) and Western blot (K) analyses of human disc tissues showing that PAR levels increase with the severity of disc degeneration. Scale bar: 100 µm. n = 3. (L) Schematic of the in vivo experiment: PAR polymer (10 µm, 2 µL) or PBS (control) was injected into rat tail discs weekly for 4 weeks; tissues were harvested at week 8 for analysis. (M) Representative T2‐weighted MRI images showing reduced signal intensity and disc height in the PAR‐injected group, indicating disc degeneration. Scale bar: 1 cm. (N–Q) Histological evaluations. (N) Representative images of disc sections using H&E, Safranin O/Fast Green, and Masson's staining. The PAR‐injected group exhibited severe matrix disruption, proteoglycan loss, and annulus fibrosus disorganization. (O–Q) Quantitative analysis showing that Pfirrmann grade and histological scores were significantly higher, and the disc height index (DHI) was lower in the PAR group, confirming PAR‐induced disc degeneration. Scale bar: 1 mm. n = 3. Data are expressed as mean ± SEM. ^*^ *p* < 0.05, ^**^ *p* < 0.01, ^***^ *p* < 0.001, and ns means not significant.

Since the unidentified cell death mechanism involved severe DNA damage and NAD^+^ depletion—hallmark features of parthanatos—we next investigated the central role of PAR polymer accumulation in this process. Immunofluorescence staining showed that PAR levels in both the cytoplasm and nucleus of NPCs significantly increased in a time‐dependent manner after 24 and 48 h of mechanical compression treatment (Figure 2A). To confirm the source and functional significance of PAR accumulation, we employed PARP inhibitors for intervention. Western blot analysis confirmed that pretreatment with AG (AG‐014699/Rucaparib, a PARP inhibitor) or ABT (ABT‐888/Veliparib, a PARP inhibitor) significantly blocked compression‐induced PAR polymer generation (Figure 2B). More importantly, functional rescue experiments demonstrated that inhibition of PARP or MIF activity markedly reversed mechanical stress‐induced NPC death (Figure 2C), proving the cytotoxic effect of PAR accumulation. Immunofluorescence results showed that mechanical stress induced upregulation of AIF, MIF, and PAR. This process was effectively inhibited by PARP or MIF inhibitors (Figure 2D). Nuclear‐cytoplasmic fractionation experiments further confirmed that compression‐induced translocation of MIF protein from the cytoplasm to the nucleus—a hallmark event in parthanatos execution [28]—was also blocked by ABT or 4‐IPP (a selective MIF inhibitor) treatment (Figure 2E). Furthermore, we examined the cellular localization of AIF and MIF using immunofluorescence. As shown in Figure 2F, mechanical stress induced a significant accumulation of AIF in the nucleus, with similar dynamics to MIF. Similarly, this nuclear translocation was effectively inhibited by ABT (PARP‐1 inhibitor) and 4‐IPP (MIF inhibitor) (Figure 2F,G). The synchronized response of both proteins to these inhibitors suggests a cooperative role in the nuclear translocation process during mechanical stress‐induced cell death. Additionally, ABT treatment prevented the compression‐induced decline in intracellular NAD^+^/NADH ratio (Figure S1E), supporting from an energy metabolism perspective that PARP inhibition maintains cellular homeostasis.

To explore the potential long‐term effects of PAR polymer in IVDD, we treated in vitro cultured NPCs with exogenous PAR. First, to confirm whether exogenous PAR could effectively penetrate the cell membrane, we synthesized Cy5.5‐labeled PAR. As shown in Figure S1F, confocal microscopy revealed that the red fluorescence of PAR‐Cy5.5 was clearly localized within the cytoplasm bounded by the F‐actin cytoskeleton, verifying its effective internalization by NPCs. Following this validation, we performed transcriptome sequencing. GSEA analysis revealed significant activation of the Hippo signaling pathway (Figure 2H), which is known to be closely associated with IVDD progression. To further clarify the key molecular events of this activation, we examined the phosphorylation status of YAP, the core effector of the Hippo pathway. Western blot analysis demonstrated that exogenous PAR treatment significantly upregulated the levels of phosphorylated YAP (p‐YAP) compared to the control group, resulting in a marked increase in the p‐YAP/Total YAP ratio (Figure S1G). This phosphorylation event typically prevents YAP nuclear translocation, thereby suppressing its transcriptional activity. Consistent with this mechanism, subsequent qPCR validation indicated that the transcriptional levels of key downstream effectors of the Hippo pathway, including *CCN2*, *MYC*, and *CCN1* were significantly suppressed (Figure 2I). Therefore, aberrant PAR accumulation may indirectly promote NPC dysfunction and the formation of a degenerative microenvironment by persistently inhibiting pro‐survival signaling pathways. To confirm the clinical relevance of PAR accumulation, we examined human intervertebral disc tissue samples. Immunofluorescence and Western blot results consistently showed significantly elevated PAR levels in severely degenerated disc tissues compared to mildly degenerated ones (Figure 2J,K), indicating a positive correlation between PAR accumulation and the severity of human IVDD. Furthermore, with increasing disc degeneration, AIF and MIF protein expression levels gradually increased, suggesting that parthanatos is involved in the progression of IVDD (Figure S1H). Finally, to validate the pathogenicity of PAR in vivo, we established a rat tail intervertebral disc PAR injection model (Figure 2L). MRI T2‐weighted image analysis revealed that the PAR injection group exhibited typical degenerative changes, manifested by significantly reduced signal intensity and disc height index (DHI) (Figure 2M). Histological staining and scoring further confirmed that PAR injection led to pathological changes of IVDD, including reduced nucleus pulposus tissue, severe proteoglycan loss, disrupted annulus fibrosus structure, and significantly increased histological scores (Figure 2N–Q). In summary, the results demonstrate that mechanical compression‐induced aberrant PAR accumulation is a central molecular event triggering parthanatos in NPCs. Concurrently, PAR may also contribute to and accelerate the pathological process of IVDD by activating pro‐degenerative signaling pathways such as the Hippo pathway.

### 2.3 Ubiquitin‐Dependent Degradation of PARG Mediates PAR Metabolism Impairment under Mechanical Compression

**FIGURE 3:**
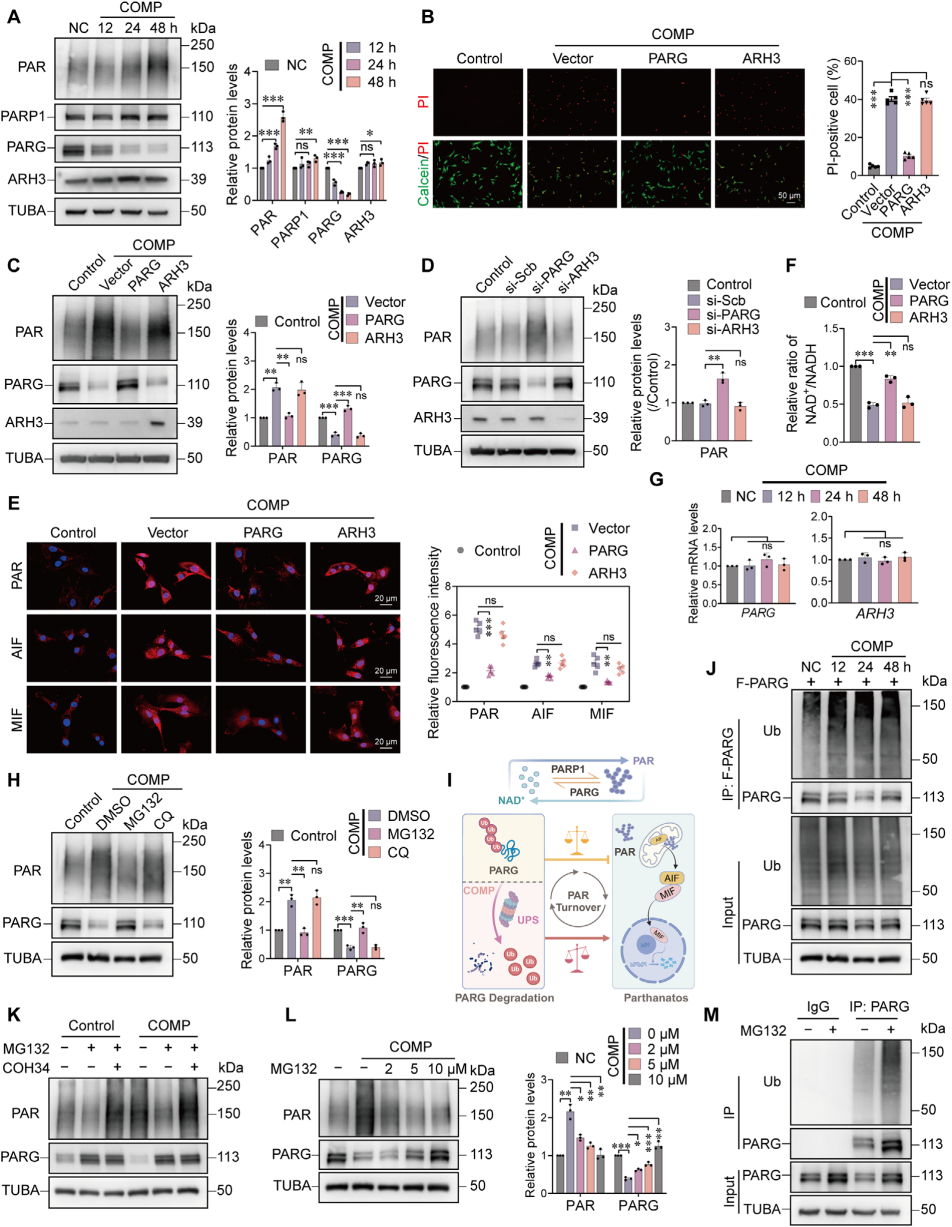
Ubiquitin‐dependent degradation of PARG mediates PAR metabolism impairment under mechanical compression. (A) Western blot analysis of PAR, PARP1, PARG, and ARH3 protein expression in NPCs following mechanical compression for 12, 24, and 48 h. n = 3. (B) Viability of NPCs overexpressing PARG or ARH3 after 48 h of compression, assessed by Calcein‐AM/PI staining. Scale bar: 50 µm. n = 5. (C) Western blot showing the effect of PARG or ARH3 overexpression on compression‐induced PAR accumulation. n = 3. (D) Effect of PARG or ARH3 knockdown on PAR levels in NPCs, as determined by Western blot. n = 3. (E) Immunofluorescence staining showing PAR, AIF, and MIF expression in compressed NPCs overexpressing PARG or ARH3. Scale bar: 20 µm. n = 5. (F) NAD^+^/NADH ratio in compressed NPCs overexpressing PARG or ARH3. n = 3. (G) RT‐qPCR analysis of *PARG* mRNA levels in NPCs subjected to compression for the indicated durations. n = 3. (H) Western blot analysis of PAR and PARG levels in compressed NPCs treated with the proteasome inhibitor MG132 (10 µm) or the lysosome inhibitor chloroquine (CQ, 20 µm) for 24 h. n = 3. (I) Schematic model illustrating the proposed mechanism by which ubiquitin‐mediated degradation of PARG disrupts PAR metabolism under mechanical compression. (J) NPCs overexpressing Flag‐PARG were subjected to compression for 12, 24, and 48 h, followed by co‐immunoprecipitation and ubiquitination assay. Western blot shows time‐dependent increase in PARG ubiquitination under compression. (K) Western blot analysis of PAR levels in compressed NPCs treated with MG132 (10 µm) with or without the PARG inhibitor COH34 (1 µm). (L) MG132 attenuated compression‐induced PAR accumulation in a dose‐dependent manner. n = 3. (M) Co‐IP assays demonstrated that MG132 treatment restored PARG protein levels under mechanical compression. Data are from at least three independent experiments and presented as mean ± SEM. ^*^ *p* < 0.05, ^**^ *p* < 0.01, ^***^ *p* < 0.001, and ns means not significant.

To elucidate the upstream mechanisms underlying the abnormal accumulation of PAR under mechanical stress, we first examined the protein expression of key regulators involved in PAR metabolism. Western blot analysis revealed that PAR levels increased significantly in a time‐dependent manner upon compression, while the protein expression of PARG decreased correspondingly (Figure 3A). Notably, the protein levels of another PAR hydrolase, ARH3, remained largely unchanged. Functional rescue assays demonstrated that overexpression of PARG significantly reversed compression‐induced cell death, whereas overexpression of ARH3 provided no protective effect (Figure 3B). Consistently, overexpressing PARG effectively reduced stress‐induced PAR accumulation, while ARH3 overexpression had no significant impact (Figure 3C). To further differentiate the roles of these two enzymes, gene knockdown experiments were conducted. The results showed that knockdown of PARG significantly exacerbated PAR accumulation under basal conditions, whereas knockdown of ARH3 had no notable effect on PAR levels (Figure 3D). These findings indicate that PARG, but not ARH3, is the dominant enzyme responsible for PAR degradation in NPCs, highlighting a cell type‐specific regulatory mechanism. Notably, overexpression of PARG markedly suppressed the execution of parthanatos, as evidenced by reduced levels of PAR, AIF, and MIF (Figure 3E), and reduced nuclear translocation of AIF and MIF (Figure S1I). Also, it prevented the compression stress‐induced decline in the NAD^+^/NADH ratio (Figure 3F). Analysis of proteins isolated from human intervertebral discs revealed that PARG protein expression levels were significantly reduced in degenerative disc tissue compared with healthy disc tissue (Figure S2A). These results further establish PARG as a core molecule maintaining PAR metabolic homeostasis in NPCs and indicate that its aberrant downregulation is a key trigger for mechanical stress‐induced parthanatos.

Although PARG protein levels decreased significantly, its mRNA expression remained unchanged after compression (Figure 3G), suggesting post‐translational regulation. We subsequently treated NPCs with the proteasome inhibitor MG132 (targeting the ubiquitin‐proteasome pathway) and the lysosome inhibitor chloroquine (CQ). Western blot results showed that MG132 treatment effectively inhibited compression‐induced PAR accumulation, whereas CQ had no such effect (Figure 3H), indicating that PARG degradation is primarily mediated by the ubiquitin‐proteasome system (UPS). We therefore propose that mechanical compression specifically promotes the ubiquitination and degradation of PARG (but not ARH3) via the UPS, thereby disrupting PAR homeostasis, leading to its toxic accumulation and ultimately triggering parthanatos (Figure 3I). To further confirm the ubiquitination of PARG, we performed co‐immunoprecipitation (Co‐IP) assays in NPCs overexpressing Flag‐PARG. The results demonstrated that the level of ubiquitin conjugated to PARG increased gradually over time following mechanical stress (Figure 3J), confirming stress‐induced ubiquitination of PARG. MG132 attenuated the mechanical stress‐induced increase in PAR levels, while the PARG inhibitor COH34 counteracted this effect of MG132 (Figure 3K). Furthermore, MG132 alleviated compression‐induced PAR accumulation in a dose‐dependent manner (Figure 3L). Co‐IP assays also indicated that MG132 treatment increased the level of PARG (Figure 3M), demonstrating that compression leads to loss of PARG function and dysregulated PAR metabolism by promoting its ubiquitination and subsequent proteasomal degradation.

### 2.4 TRIM25 Targets PARG to Promote its Ubiquitin‐Dependent Degradation under Compression

**FIGURE 4:**
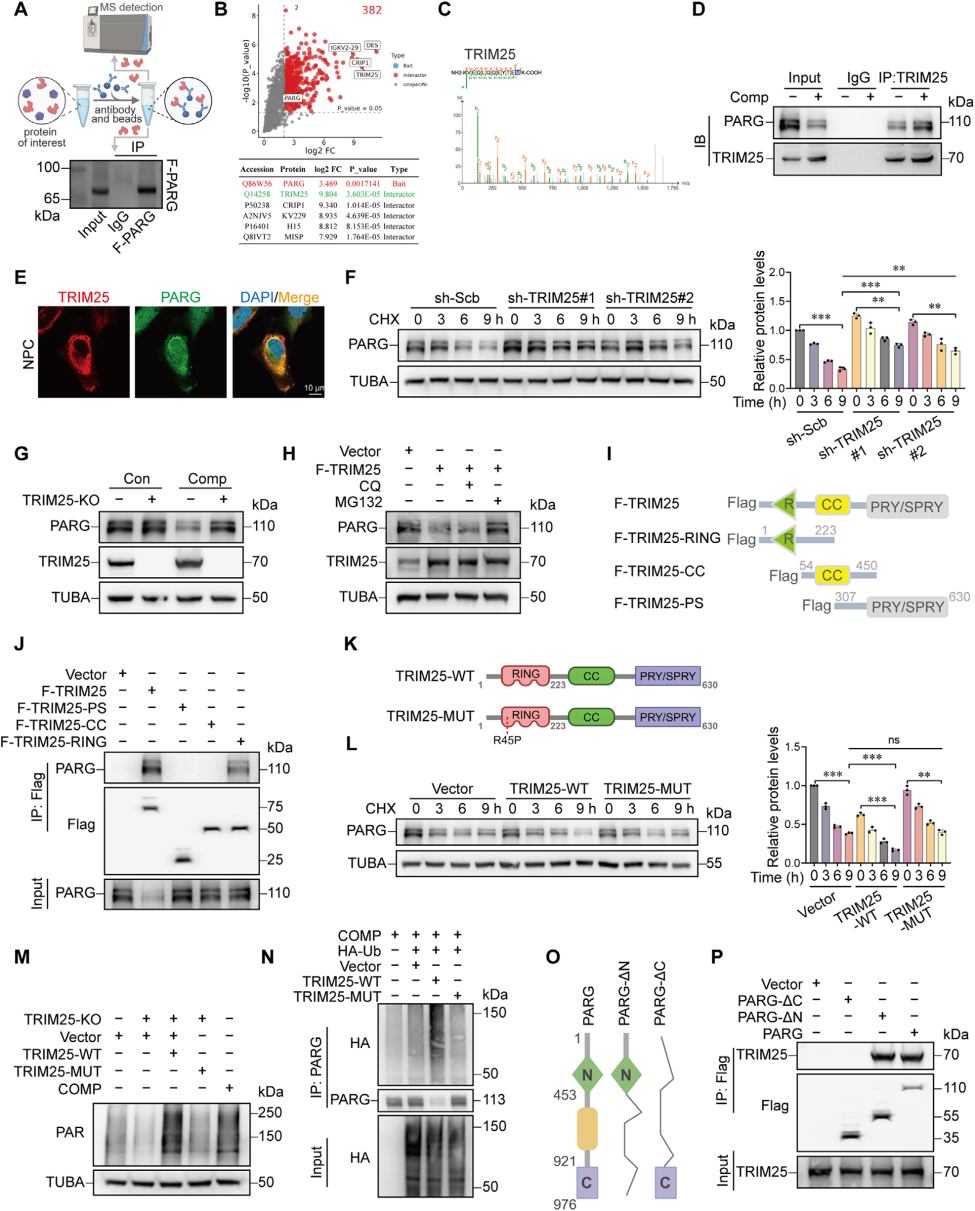
TRIM25 targets PARG to promote its ubiquitin‐dependent degradation under compression. (A–B) Co‐immunoprecipitation coupled with mass spectrometry (Co‐IP/MS) using PARG as bait. Volcano plot of differentially bound proteins identifies TRIM25 as one of the most significant interactors. (C) MS/MS spectrum of TRIM25. (D) Co‐IP assays using an anti‐TRIM25 antibody showed enhanced interaction between TRIM25 and PARG under mechanical compression. (E) Immunofluorescence staining revealed significant cytoplasmic colocalization of TRIM25 and PARG. Scale bar: 10 µm. (F) Cycloheximide (CHX, 50 µg/mL) chase assay demonstrated that TRIM25 knockdown delayed PARG protein degradation. n = 3. (G) Western blot analysis of PARG protein levels in TRIM25‐knockdown NPCs under mechanical compression. (H) Overexpression of TRIM25 reduced PARG protein levels, an effect reversed by the proteasome inhibitor MG132 (10 µm) but not by the lysosome inhibitor CQ (20 µm). (I) Schematic of TRIM25 truncation mutants, including RING, coiled coil (CC), and PRY/SPRY (PS) domains. (J) Co‐IP assays indicated that the fragment containing the RING domain of TRIM25 binds to PARG, while the CC and PS mutants do not. (K) Schematic of the TRIM25 RING domain point mutant (R45P), which lacks E3 ligase activity. (L) CHX chase assay showed that overexpression of wild‐type (WT) TRIM25, but not the R45P mutant (Mut), accelerated PARG degradation. n = 3. (M) Western blot analysis showed that reconstitution with TRIM25‐WT, but not TRIM25‐Mut, promoted PAR accumulation in TRIM25‐knockout NPCs. The compression group served as a positive control. (N) Co‐IP assays under mechanical compression confirmed that TRIM25‐WT, but not the mutant, promoted PARG ubiquitination and degradation. (O) Schematic of PARG truncation constructs, including N‐terminal and C‐terminal fragments. (P) Co‐IP assays indicated that full‐length PARG and its N‐terminal fragment bound to TRIM25, while the C‐terminal fragment did not, suggesting that TRIM25 binds to the N‐terminal region of PARG. Data are from at least three independent experiments and presented as mean ± SEM. ^*^ *p* < 0.05, ^**^ *p* < 0.01, ^***^ *p* < 0.001, and ns means not significant.

Building on the role of the UPS, we next sought to identify the key molecule mediating PARG degradation. Co‐immunoprecipitation coupled with mass spectrometry (Co‐IP/MS) analysis of PARG revealed that among all potential interacting proteins, TRIM25 exhibited the most significant binding (Figure 4A–C). Co‐IP assays further confirmed the interaction between TRIM25 and PARG, which was enhanced upon compression (Figure 4D). Immunofluorescence staining revealed that PARG was distributed in both the nucleus and cytoplasm (Figure 4E), consistent with the expression of alternatively spliced isoforms that localize to different compartments [29].

To validate the specific subcellular distribution of PARG isoforms, we performed nuclear/cytoplasmic fractionation assays. As shown in Figure S2B, the full‐length PARG isoform was enriched in the nucleus, whereas shorter isoforms were predominantly detected in the cytoplasm. Importantly, TRIM25 was predominantly localized in the cytoplasm, where it exhibited significant co‐localization with these cytoplasmic PARG isoforms (Figure 4E; Figure S2B). To investigate whether TRIM25 regulates PARG stability, we first examined its effect on PARG protein half‐life. A cycloheximide (CHX) chase assay demonstrated that the degradation rate of PARG was significantly slowed in TRIM25‐knockdown NPCs (Figure 4F). Correspondingly, TRIM25 knockout effectively reversed the mechanical stress‐induced downregulation of PARG protein (Figure 4G), providing direct causal evidence that compression‐induced PARG degradation is driven by TRIM25. Conversely, overexpression of TRIM25 reduced PARG protein levels, an effect that could be blocked by MG132 (Figure 4H), indicating that TRIM25 regulates PARG degradation via the proteasomal pathway.

We next dissected the structural basis of the TRIM25‐PARG interaction. Using a series of TRIM25 truncated mutants (Figure 4I), Co‐IP assays revealed that only the fragment containing the RING domain could bind PARG (Figure 4J), suggesting that the RING domain is necessary for the interaction. To validate the functional role of TRIM25's E3 ligase activity in this process, we generated a mutant (TRIM25‐Mut) with a key catalytic site mutation (R45P) in the RING domain (Figure 4K). Functional assays showed that overexpression of wild‐type TRIM25 (WT) accelerated PARG degradation, whereas the mutant (Mut) had no such effect (Figure 4L). Reconstitution of TRIM25‐knockout cells with TRIM25‐WT promoted PAR accumulation, while reconstitution with the Mut did not (Figure 4M). Importantly, under mechanical stress, overexpression of TRIM25‐WT significantly enhanced PARG ubiquitination and promoted its degradation, while overexpression of the Mut had no effect (Figure 4N). To identify the region on PARG responsible for TRIM25 binding, we constructed N‐terminal and C‐terminal truncated PARG mutants (Figure 4O). The results indicated that full‐length PARG and the N‐terminal fragment could bind TRIM25, whereas the C‐terminal fragment could not (Figure 4P), suggesting that TRIM25 specifically recognizes the N‐terminal region of PARG. To pinpoint the specific amino acid residue responsible for this interaction, we further generated PARG point mutants, including K115A and K451A. As shown in Figure S2C, Co‐IP assays demonstrated that the K115A mutation completely abolished the interaction with TRIM25, whereas the K451A mutant retained binding capacity. These findings unequivocally identify Lysine 115 as the critical site for ubiquitination of PARG by TRIM25. In summary, under mechanical stress, TRIM25 directly binds the Lysine 115 residue at the N‐terminal region of PARG via its RING domain and promotes the ubiquitination and proteasomal degradation of PARG, ultimately leading to dysregulated PAR metabolism in NPCs.

### 2.5 Elevated TRIM25 Exacerbates DNA Damage in NPCs under Compression

**FIGURE 5:**
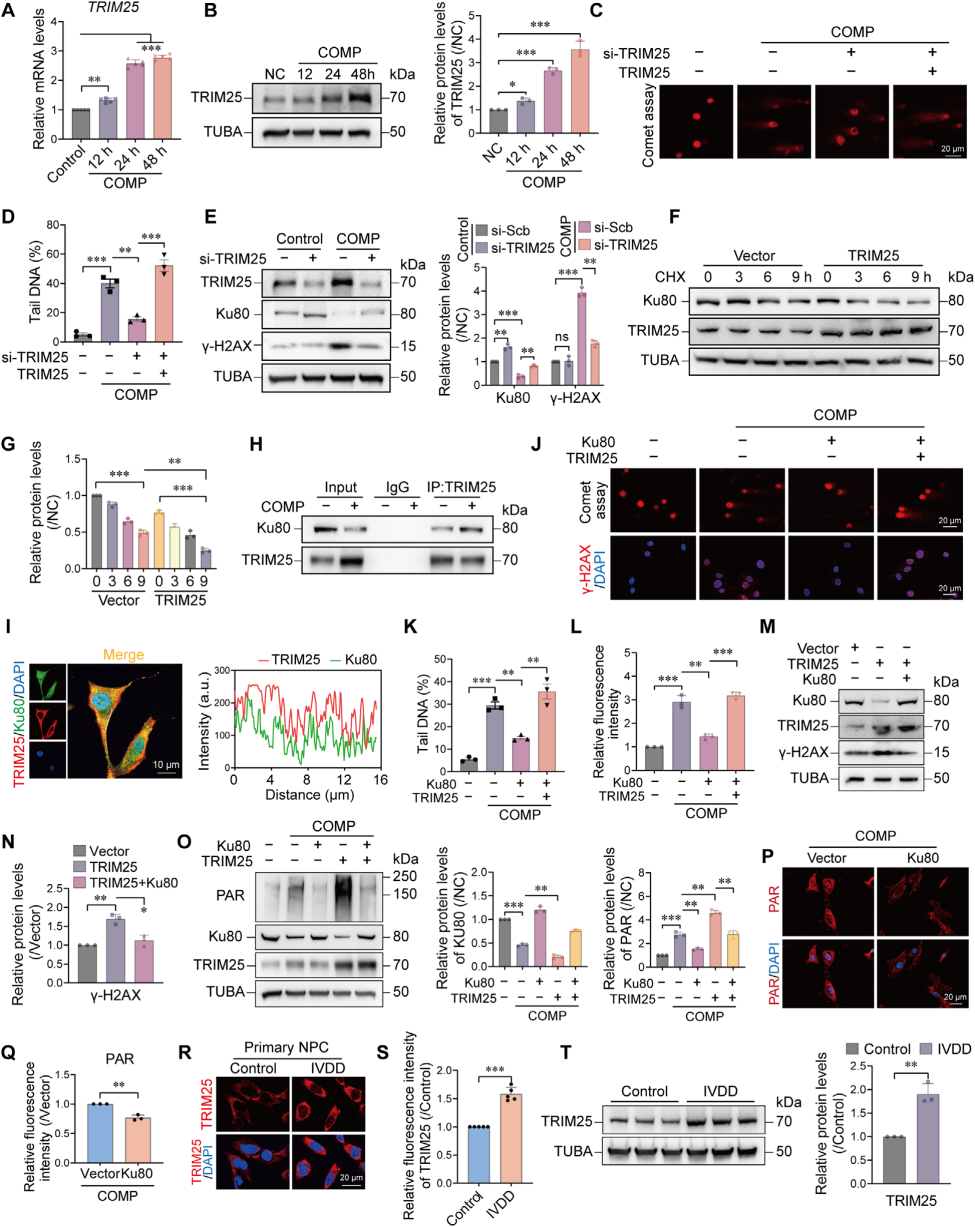
Elevated TRIM25 exacerbates DNA damage in NPCs under compression. (A) RT‐qPCR analysis showing time‐dependent upregulation of *TRIM25* mRNA in NPCs following mechanical compression for 12, 24, and 48 h. n = 5. (B) Western blot demonstrating increased TRIM25 protein expression under mechanical compression in a time‐dependent manner. n = 3. (C–D) Comet assays revealed that TRIM25 knockdown alleviated, while TRIM25 overexpression aggravated, compression‐induced DNA damage in NPCs. Scale bar: 20 µm. n = 3. (E) Western blot showed that compression‐induced TRIM25 upregulation coincided with decreased Ku80 protein levels; TRIM25 knockdown restored Ku80 expression. n = 3. (F–G) CHX‐based protein stability assays indicated that TRIM25 overexpression accelerated Ku80 degradation. n = 3. (H) Co‐immunoprecipitation assays demonstrated enhanced interaction between TRIM25 and Ku80 after mechanical compression. (I) Immunofluorescence staining confirmed intracellular colocalization of TRIM25 (red) and Ku80 (green). Scale bar: 10 µm. (J–L) Comet assays and γ‐H2AX immunofluorescence showed that Ku80 overexpression attenuated compression‐induced DNA damage, an effect reversed by co‐overexpression of TRIM25. Scale bar: 20 µm. n = 3. (M–N) Western blot analysis indicated that TRIM25 overexpression reduced Ku80 and increased γ‐H2AX levels, which were rescued by Ku80 co‐expression. n = 3. (O) Western blot analysis showing that Ku80 overexpression reduced compression‐induced PAR accumulation, and this effect was suppressed by TRIM25 co‐overexpression. n = 3. (P–Q) Immunofluorescence staining confirmed that Ku80 overexpression inhibited compression‐induced PAR accumulation in NPCs. Scale bar: 20 µm. n = 3. (R–S) Immunofluorescence of human disc tissues showed higher TRIM25 expression in degenerated than in healthy discs. Scale bar: 20 µm. n = 5. (T) Western blot of primary NP cells isolated from human normal and degenerated discs confirmed that TRIM25 protein levels increased with disc degeneration severity. n = 3. Data are presented as mean ± SEM. ^*^ *p* < 0.05, ^**^ *p* < 0.01, ^***^ *p* < 0.001, and ns means not significant.

Given the established role of TRIM25 in DNA damage across several pathological models [30, 31], it is necessary to further elucidate its role in DNA damage under compression. We first examined the expression pattern of TRIM25 under compression. Consistent results from RT‐qPCR and Western blot analysis revealed that mechanical compression induced a significant, time‐dependent upregulation of TRIM25 at both the mRNA and protein levels (Figure 5A,B), identifying TRIM25 as a key compression‐responsive gene. To elucidate the upstream mechanotransduction signaling responsible for this induction, we investigated the role of intracellular calcium influx, a primary response to mechanical stress. We found that treatment with the specific calcium chelator BAPTA‐AM effectively abolished the compression‐induced upregulation of TRIM25 (Figure S2D,E), indicating that mechanical stress triggers TRIM25 expression via a calcium‐dependent signaling pathway. To assess the impact of TRIM25 on DNA damage, we evaluated the effects of its gain‐ and loss‐of‐function using the comet assay and γ‐H2AX assay. The results demonstrated that TRIM25 knockdown significantly alleviated mechanical stress‐induced DNA damage, while its overexpression exacerbated DNA damage (Figure 5C,D; Figure S2F). Based on prior reports [32], we hypothesized that TRIM25 might influence DNA damage levels by regulating the stability of the DNA repair protein Ku80. Notably, mechanical stress reduced Ku80 protein levels, an effect that was reversed by TRIM25 knockdown (Figure 5E). A CHX chase assay further confirmed that TRIM25 overexpression significantly accelerated Ku80 protein degradation (Figure 5F,G). Mechanistically, co‐immunoprecipitation assays showed that mechanical stress enhanced the interaction between TRIM25 and Ku80 and their co‐localization within NPCs (Figure 5H,I), suggesting that TRIM25 may target Ku80 as an E3 ubiquitin ligase. To verify the specificity of this regulation within the DDR network, we further examined key kinases ATM and ATR. TRIM25 overexpression suppressed the phosphorylation of ATM and its target CHK2 upon DNA damage induction, while leaving the ATR‐CHK1 axis largely unaffected (Figure S2G). This selective suppression aligns with the specific targeting of Ku80, a core component of DSB repair, rather than ssDNA sensing pathways.

To confirm the functional impact of TRIM25‐mediated Ku80 depletion on DNA repair capacity, we assessed cell survival under treatment with the DSB‐inducer Etoposide. TRIM25‐overexpressing cells exhibited significantly reduced viability and hypersensitivity to Etoposide (Figure S2H,I), indicating a compromised NHEJ pathway. To validate this functional relationship, a series of rescue experiments was performed. Ku80 overexpression effectively attenuated compression‐induced DNA damage in NPCs, and this protective effect was completely abolished by co‐overexpression of TRIM25 (Figure 5J–L). Further confirmation showed that TRIM25 overexpression reduced Ku80 protein levels and increased γ‐H2AX expression, both of which were rescued by Ku80 co‐expression (Figure 5M,N). Moreover, Ku80 overexpression also mitigated compression‐induced PAR accumulation, and this effect was similarly reversed by TRIM25 overexpression (Figure 5O–Q), indicating that TRIM25 partially promotes parthanatos by degrading Ku80 and aggravating DNA damage response. Additionally, we investigated the clinical relevance of TRIM25. The results showed that TRIM25 expression was significantly higher in degenerated human discs compared to healthy controls (Figure 5R–T; Figure S2J), and positively correlated with the severity of degeneration, underscoring a critical role for TRIM25 in IVDD pathophysiology. Collectively, upregulation of TRIM25 not only induces parthanatos via PARG degradation but also impairs DNA damage repair capacity by promoting the degradation of Ku80, thereby exacerbating genomic instability and abnormal PAR accumulation under compression.

### 2.6 TRIM25 Knockdown Alleviates Compression‐Induced Parthanatos and Inflammation in NPCs

**FIGURE 6:**
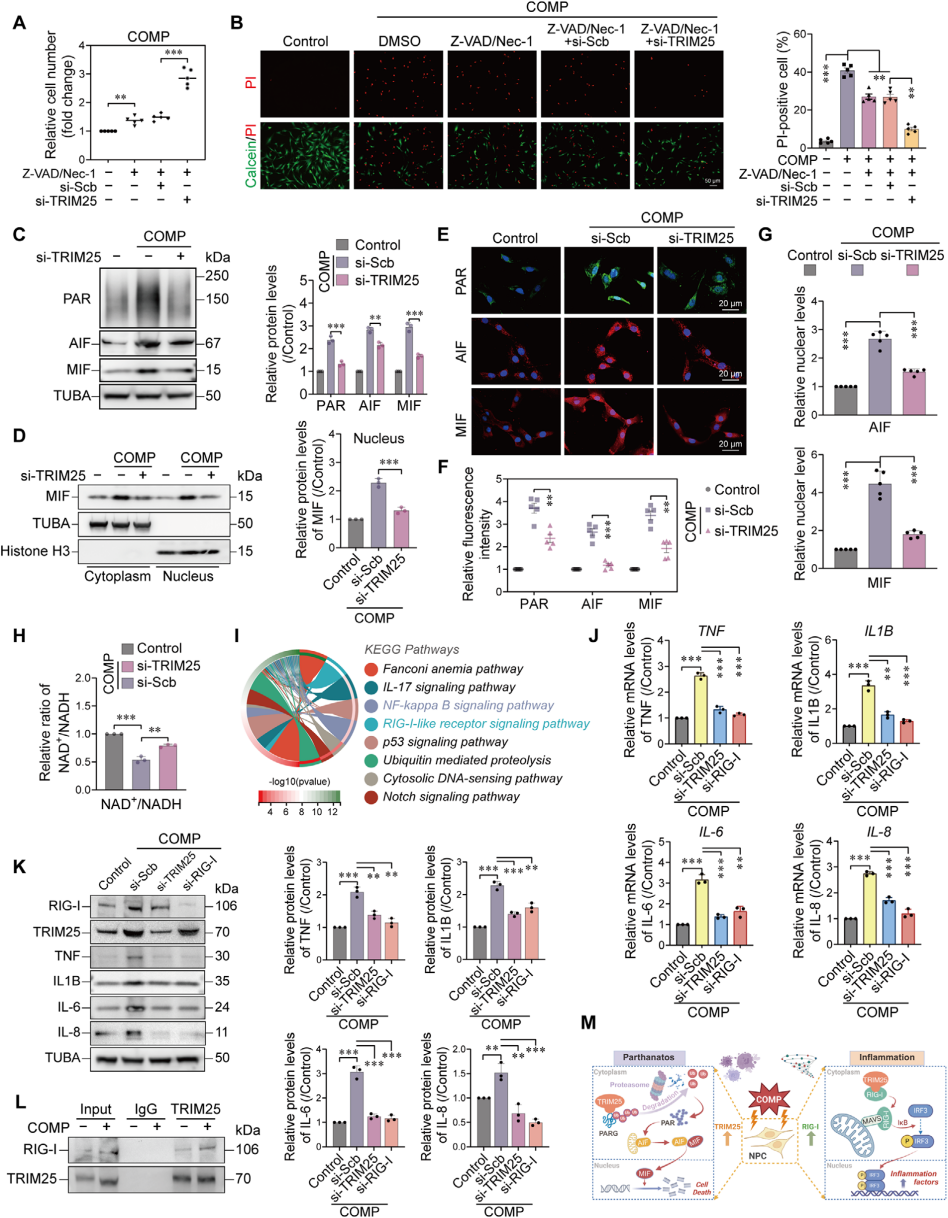
TRIM25 knockdown alleviates compression‐induced parthanatos and inflammation in NPCs. (A) Cell counting assays showed that combined treatment of Z‐VAD (20 µm) and Nec‐1 (20 µm) partially alleviated compression‐induced death of NPCs, and this protective effect was enhanced by TRIM25 knockdown. n = 5. (B) Viability staining with Calcein‐AM/PI confirmed that TRIM25 knockdown significantly reduced the death of NPCs under mechanical compression, compared with the combined treatment of Z‐VAD and Nec‐1. Scale bar: 50 µm. n = 5. (C) Western blot analysis indicated that TRIM25 knockdown suppressed compression‐induced PAR accumulation and upregulation of AIF and MIF. n = 3. (D) Cytosolic/nuclear fractionation followed by Western blot revealed that TRIM25 knockdown inhibited compression‐induced nuclear translocation of MIF (histone H3 served as nuclear loading control). n = 3. (E–G) Immunofluorescence staining demonstrated that TRIM25 knockdown markedly attenuated the upregulation of PAR, AIF, and MIF in compressed NPCs, and inhibited the nuclear translocation of MIF and AIF. Scale bar: 20 µm. n = 5. (H) TRIM25 knockdown ameliorated the compression‐induced decline in the NAD^+^/NADH ratio. n = 3. (I) RNA sequencing and KEGG pathway analysis of compressed NPCs identified significant enrichment of the RIG‐I‐like receptor signaling pathway. (J–K) RT‐qPCR (J) and Western blot (K) analyses showed that mechanical compression upregulated the expression of TNF, IL1B, IL‐6, and IL‐8, which was suppressed by knockdown of either TRIM25 or RIG‐I. n = 3. (L) Co‐immunoprecipitation assays indicated enhanced interaction between TRIM25 and RIG‐I under mechanical compression. (M) Schematic model illustrating that mechanical compression triggers both parthanatos and RIG‐I‐mediated inflammation through upregulation of TRIM25. Data are expressed as mean ± SEM. ^*^ *p* < 0.05, ^**^ *p* < 0.01, ^***^ *p* < 0.001, and ns means not significant.

To evaluate the protective effect of TRIM25 knockdown under mechanical stress, we first assessed the overall impact of TRIM25 modulation on NPC viability. Although treatment with Z‐VAD/Nec‐1 partially alleviated compression‐induced cell death, combined TRIM25 knockdown resulted in a stronger protective effect (Figure 6A,B), suggesting that TRIM25 primarily regulates a caspase‐ and MLKL‐independent cell death pathway. Western blot analysis showed that TRIM25 knockdown effectively suppressed mechanical stress‐induced PAR accumulation and the upregulation of AIF and MIF expression (Figure 6C). Nuclear‐cytoplasmic fractionation assays further demonstrated that TRIM25 knockdown prevented the nuclear translocation of MIF (Figure 6D). Consistent with these findings, immunofluorescence staining revealed that TRIM25 knockdown significantly reduced PAR levels, decreased AIF and MIF expression, and diminished nuclear localization of AIF and MIF (Figure 6E–G). Moreover, TRIM25 knockdown ameliorated the compression‐induced decline in the NAD^+^/NADH ratio (Figure 6H), confirming its protective role at the level of cellular energy metabolism. This may be due to the increased efficiency of PAR metabolism.

KEGG pathway analysis of compression‐treated NPCs indicated significant activation of the RIG‐I‐like receptor signaling pathway (Figure 6I). We subsequently verified that RIG‐I expression increased in a time‐dependent manner after compression treatment (Figure S3A–C). Given previously reported immunoregulatory functions of TRIM25 [33, 34], we hypothesized that it may contribute to this process. Further experiments confirmed that mechanical compression upregulated the expression of multiple inflammatory factors (TNF, IL1B, IL‐6, and IL‐8) in NPCs, while knockdown of TRIM25 or RIG‐I effectively inhibited the cellular levels of these inflammatory factors (Figure 6J,K). To verify whether these factors were actively secreted into the microenvironment, we analyzed the cell culture supernatant using ELISA. As expected, mechanical stress led to a dramatic increase in the extracellular concentrations of TNF, IL1B, IL‐6, and IL‐8. Importantly, knockdown of TRIM25 and RIG‐I significantly inhibited this secretion peak (Figure S3D). Compression also significantly increased IFNB1 mRNA levels and IFN‐β secretion, and knockdown of TRIM25 or RIG‐I effectively reversed these changes (Figure S3E,F). Furthermore, compression enhanced the protein interaction and intracellular colocalization between TRIM25 and RIG‐I (Figure S3G,H). This indicates that elevated TRIM25 also contributes to the inflammatory response under compression via activation of the RIG‐I signaling pathway. Co‐immunoprecipitation assays confirmed that under compression, the interaction between TRIM25 and RIG‐I was enhanced (Figure 6L). Thus, beyond its role in promoting PAR toxicity and parthanatos, upregulation of TRIM25 also exacerbates inflammation through activation of the RIG‐I pathway. Knockdown of TRIM25 concurrently attenuated both cell death and inflammatory responses, significantly improving NPC survival under mechanical stress (Figure 6M).

Notably, given the observed accumulation of DNA damage and release of inflammatory factors, we further explored the potential role of TRIM25 in cellular senescence. As shown in Figure S3I–K, mechanical compression significantly upregulated the expression of senescence markers (CDKN2A and CDKN1A) and increased SA‐β‐gal activity, while knockdown of TRIM25 effectively reversed these effects. Although this differs from the acute cell death pathway described above, these findings suggest that TRIM25 may simultaneously drive NPC senescence by bridging the DNA damage response and the inflammatory microenvironment, thereby accelerating intervertebral disc degeneration through multiple mechanisms. This potential TRIM25‐senescence axis provides a compelling direction for future mechanistic studies.

### 2.7 In vivo TRIM25 Silencing or PARG Overexpression Alleviates Compression‐Induced IVDD in Rats

**FIGURE 7:**
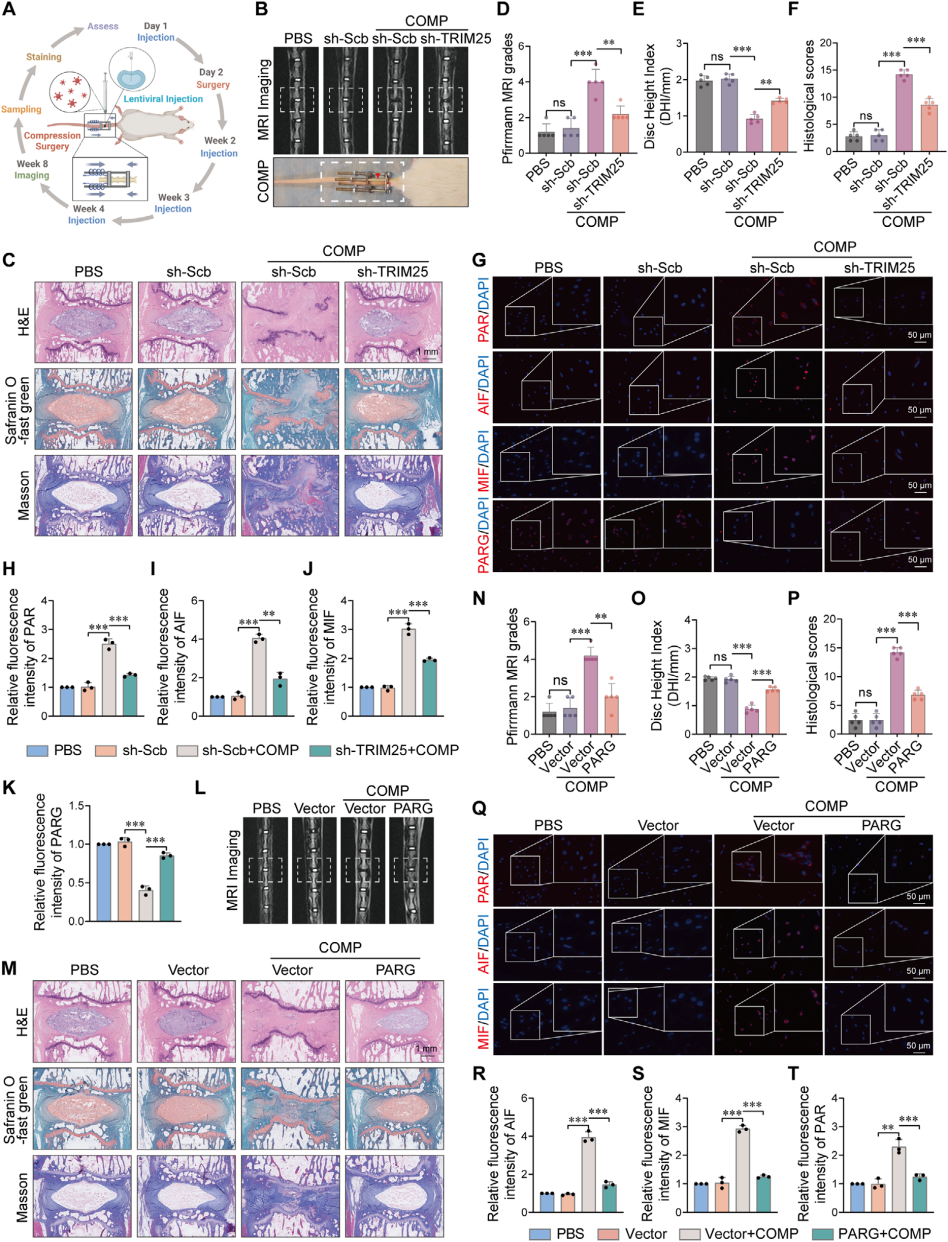
In vivo TRIM25 silencing or PARG overexpression alleviates compression‐induced IVDD in rats. (A) Schematic of the rat tail disc degeneration model. An external compression device was installed, accompanied by weekly local injections of lentivirus carrying shTRIM25 or oePARG (1 × 10^8^ TU/mL, 2 µL) or PBS control for 4 weeks. MRI was performed at week 8, and disc tissues were harvested for histological analysis. (B) Representative T2‐weighted MRI images showing decreased signal intensity in compressed discs, which was partially restored by shTRIM25 treatment. The lower panel shows the external compression device. (C–F) Histological evaluations. (C) Representative images of disc sections using H&E, Safranin O/Fast Green, and Masson's staining. (D–F) Quantitative IVDD assessments based on Pfirrmann grade, disc height index (DHI), and histological scoring. Scale bar: 1 mm. n = 3. (G–K) Immunofluorescence staining of disc tissues revealed that mechanical compression increased PAR, AIF, and MIF levels and decreased PARG expression, effects that were reversed by shTRIM25 lentiviral injection. Scale bar: 50 µm. n = 3. (L) Representative T2‐weighted MRI images demonstrating that PARG overexpression alleviated compression‐induced rat disc degeneration. (M) Histological staining showed better preservation of nucleus pulposus and annulus fibrosus structures in the oePARG group compared to the compression group. Scale bar: 1 mm. (N–P) Quantitative evaluation of disc degeneration using Pfirrmann score, DHI, and histological score in the compression and compression + oePARG groups. n = 3. (Q‐T) Immunofluorescence staining indicated that PARG overexpression suppressed compression‐induced PAR accumulation and upregulation of AIF and MIF, suggesting inhibition of parthanatos. Scale bar: 50 µm. n = 3. Data are presented as mean ± SEM. ^*^ *p* < 0.05, ^**^ *p* < 0.01, ^***^ *p* < 0.001, and ns means not significant.

To validate the pathological significance and therapeutic potential of the TRIM25–PARG axis in vivo, we established a mechanical compression‐induced IVDD model in rat caudal vertebrae. Mechanical stress was applied using crossed Kirschner wires and compression springs [27], with corresponding interventions administered via local lentiviral injection (Figure 7A). After 8 weeks, T2‐weighted MRI images showed that the signal intensity and water content of the intervertebral disc in the compression group were significantly reduced, indicating the occurrence of IVDD. In contrast, co‐treatment with sh‐TRIM25 lentivirus markedly restored disc signal intensity (Figure 7B). Histopathological examination further demonstrated that mechanical stress resulted in structural disruption of the nucleus pulposus, proteoglycan loss, and disorganized collagen architecture. These pathological changes were significantly ameliorated in the sh‐TRIM25 treatment group (Figure 7C–F), suggesting that TRIM25 knockdown alleviates compression‐induced IVDD in rats. Consistently, immunofluorescence analysis revealed that mechanical compression caused a substantial loss of Collagen‐II and Aggrecan, confirming matrix degradation; however, sh‐TRIM25 treatment effectively preserved these components (Figure S4A). Immunofluorescence staining showed elevated levels of PAR, AIF, and MIF, along with reduced PARG expression and increased IL1B accumulation in compression‐treated discs, confirming the activation of parthanatos and inflammation under mechanical load (Figure 7G–K; Figure S4B). However, sh‐TRIM25 treatment effectively reversed these molecular alterations (Figure 7G–K; Figure S4B), suppressing parthanatos activation and inflammatory responses in vivo.

We further evaluated the therapeutic effect of PARG overexpression. MRI analysis indicated that lentivirus‐mediated PARG overexpression attenuated the severity of compression‐induced IVDD (Figure 7L). Histological staining demonstrated better‐preserved disc structure, higher proteoglycan content, and more organized annulus fibrosus in the PARG‐overexpression group (Figure 7M). Quantitative evaluations confirmed significant improvements in Pfirrmann grade, disc height index (DHI), and histological scores following PARG overexpression (Figure 7N–P). Similarly, while mechanical stress depleted matrix proteins, PARG overexpression significantly restored the fluorescence intensity of Collagen‐II and Aggrecan (Figure S4C). Immunofluorescence analysis further corroborated that PARG overexpression suppressed mechanical stress‐induced PAR accumulation and parthanatos activation (Figure 7Q–T). In summary, our in vivo findings demonstrate that either silencing TRIM25 or overexpressing PARG effectively mitigates mechanical stress‐induced intervertebral disc degeneration through inhibition of PAR accumulation and parthanatos activation. These results not only confirm the central role of the TRIM25–PARG pathway in compression‐induced IVDD but also highlight its potential as a therapeutic target.

## 3 Discussion

Abnormal mechanical loading is a major contributor to IVDD, yet how mechanical stress coordinates multiple downstream pathological processes remains poorly understood. Our study identifies the E3 ubiquitin ligase TRIM25 as a critical transducer of mechanical signals that drives IVDD progression through multiple mechanisms. Specifically, mechanical compression induces TRIM25 upregulation and disrupts poly (ADP‐ribose) (PAR) metabolism via ubiquitin‐mediated degradation of PARG, leading to parthanatos, a form of programmed cell death. Concurrently, TRIM25 facilitates the degradation of Ku80, impairing the DNA damage repair capacity and further promoting abnormal PAR accumulation. In addition, TRIM25 enhances the inflammatory response in NPCs under mechanical stress by activating the RIG‐I signaling pathway. These findings systematically elucidate the central regulatory role of TRIM25 in IVDD and provide a theoretical foundation for future therapeutic strategies targeting TRIM25.

Elucidating the precise nature of cell death is a prerequisite for understanding IVDD pathology. A pivotal observation in our study was the inability of the pan‐caspase inhibitor Z‐VAD to completely prevent compression‐induced cell death, despite the effective blockade of Caspase‐3 and GSDMD processing. This “partial rescue” phenomenon led us to identify a “Double‐Hit” mechanism: the concomitant activation of PANoptosis (evidenced by elevated GSDMD‐N and p‐MLKL) and a distinct, caspase‐independent lethal program. Even combined inhibition of caspases and RIPK1 failed to fully restore cell viability, pointing to the involvement of parthanatos as the second, fatal hit driven by metabolic collapse. Although parthanatos is known to be triggered under conditions of DNA damage and closely associated with abnormal accumulation of PAR [35], its specific role in IVDD has remained unclear. This study provides, for the first time, a complete mechanistic link demonstrating that an imbalance between PAR generation and degradation culminates in cytotoxic stress and parthanatos in a compression‐induced IVDD model. Notably, the biological functions and regulatory mechanisms of PAR—an epigenetically relevant byproduct in the context of DNA damage—have not been previously elucidated in musculoskeletal disorders. We further revealed that impaired PAR degradation is not mediated by ARH3, but rather results from reduced PARG expression, likely reflecting cell type‐specific regulatory patterns. Biochemically, this reliance on PARG may be attributed to its distinct kinetics as the primary glycohydrolase responsible for rapid bulk degradation. In contrast, ARH3 exhibits lower catalytic efficiency and specifically targets terminal serine‐ADP‐ribose moieties, which likely renders it insufficient to compensate for PARG depletion under acute stress [36]. Interestingly, while substrate accumulation typically triggers a compensatory upregulation of metabolic enzymes, we observed a paradoxical decline in PARG protein levels. Our data resolved this anomaly by demonstrating that the decrease in PARG occurred not at the transcriptional level, but through UPS‐mediated protein degradation. Using Co‐IP/MS, we identified TRIM25 as the key E3 ligase directly regulating PARG stability. Regarding the spatial context of this regulation, previous immunocytochemistry, immunoblots, and PARG enzyme activity assays have shown that cytoplasmic isoforms contribute the majority of cellular PARG activity, in the presence or absence of genotoxic stress [29]. This suggests that TRIM25‐mediated ubiquitination specifically targets this highly active cytoplasmic pool, thereby efficiently compromising the cell's PAR‐hydrolyzing capacity. Crucially, we confirmed that mechanical compression acts as a potent upstream stimulus that significantly upregulates TRIM25 via a calcium‐dependent signaling pathway, and functional rescue experiments verified that this upregulation is the direct cause of PARG loss. This clarifies the “Compression/TRIM25/PARG” axis and uncovers a novel parthanatos activation pathway mediated by a TRIM family protein. TRIM proteins play pivotal roles in cellular stress responses and substrate ubiquitination [37]. Mounting evidence implicates TRIM family members in various musculoskeletal disorders, including IVDD [24, 38]. For instance, TRIM32 exacerbates inflammatory cytokine‐induced apoptosis in NPCs by ubiquitinating AXIN1 [26]; TRIM16 is upregulated in degenerated disc tissues and correlates positively with oxidative stress levels [39]; and overexpression of TRIM14 mimics the regulatory effects of TNF on NPC survival and apoptosis [40]. Our study is the first to demonstrate that TRIM25 induces the death of NPCs by mediating PARG degradation and disrupting PAR metabolism. These findings, together with previous reports, underscore the crucial regulatory roles of TRIM family proteins in IVDD.

Our findings on TRIM25‐mediated parthanatos add a novel, mechanosensitive dimension to the growing understanding of DNA damage in IVDD pathogenesis. Recent elegant studies have highlighted other critical layers of this complex process. For instance, the abnormal accumulation of intracellular and extracellular nucleic acids has been identified as a key driver of chronic inflammation in IVDD, suggesting that DNA damage and its sequelae extend beyond the nucleus pulposus cell itself to shape the degenerative microenvironment [41]. Furthermore, epigenetic regulation, such as N6‐methyladenosine (m6A) modification of circular RNAs (e.g., circGPATCH2L), can influence DNA damage accumulation and apoptosis through proteins like TRIM28, revealing a sophisticated post‐transcriptional layer of control [21]. Additionally, the dysfunction of the cytoplasmic DNA sensor STING and its autophagic degradation in senescent NPCs illustrates how impaired clearance of DNA damage signals can perpetuate inflammation and accelerate degeneration [42]. These studies collectively underscore that IVDD‐associated DNA damage is a multi‐faceted event, triggered by various stressors (e.g., mechanical load, senescence, metabolic stress) and resolved or exacerbated through diverse molecular nodes (e.g., nucleic acid scavenging, non‐coding RNA networks, innate immune sensors). Our work identifies mechanical compression as a potent upstream inducer and TRIM25‐mediated ubiquitination of PARG as a specific, decisive mechanism that disrupts PAR metabolism and commits cells to parthanatos. This pathway operates alongside, and may interact with, these other mechanisms, painting a more integrated picture of how genomic instability converges with cell death and inflammation to drive disc degeneration.

Given the key role of TRIM25 in affecting genome stability and various inflammatory pathological processes [43], we hypothesized that it may also exert pleiotropic pathogenic functions in IVDD. Beyond its regulation of PARG, we found that TRIM25 impairs DNA repair capacity by promoting Ku80 degradation. This dual mechanism—simultaneously compromising DNA repair and facilitating parthanatos—may reflect a cellular “point of no return” decision under severe stress. Furthermore, we demonstrated that compression‐induced activation of the RIG‐I signaling pathway and subsequent inflammatory responses are both dependent on TRIM25 upregulation, consistent with its established role in innate immunity [33]. This highlights an intriguing biochemical versatility of TRIM25: whereas it facilitates non‐degradative regulatory ubiquitination (typically K63‐linked) to activate the RIG‐I signalosome [23], our data demonstrate that it concurrently drives the ubiquitin‐mediated proteasomal degradation of PARG and Ku80. This suggests that TRIM25 operates through distinct ubiquitin topologies—likely canonical degradative chains vs. regulatory chains—depending on the substrate context [44]. This multifunctional regulatory capacity enables TRIM25 to integrate mechanical stress signals and coordinate multiple downstream events, including genomic instability, loss of proteostasis, and formation of an inflammatory microenvironment, collectively driving IVDD progression. TRIM25 can target both PARG and Ku80 simultaneously, raising an important question: are these interactions mutually exclusive or synergistic? Our localization data indicate that the interaction sites between TRIM25 and PARG are distinct from the reported TRIM25‐Ku80 interaction interface [32]. This spatial separation of binding sites suggests that TRIM25 may bind to its substrates through independent domains, favoring a sequential or parallel, non‐competitive ubiquitination event model rather than the formation of a stable ternary complex. This structure allows TRIM25 to effectively coordinate the simultaneous dysregulation of PAR metabolism and DNA repair under mechanical stress, thereby exacerbating cellular dysfunction and IVDD (Figure S5). Future studies will require detailed kinetic and structural analyses to fully elucidate the temporal regulation and potential hierarchical relationships of TRIM25 substrate selection. From a translational perspective, targeting TRIM25 holds promise for concurrently modulating multiple pathogenic pathways, offering a potential therapeutic advantage over strategies focused on single processes such as apoptosis or specific inflammatory factors. These findings provide a strong rationale for developing disease‐modifying therapies for IVDD driven by multiple etiologies, particularly early degeneration driven by abnormal mechanical loading.

Previous studies have indicated that persistently elevated levels of PAR in cerebrospinal fluid contribute to the progression of neurodegenerative diseases [15, 45]. To investigate the potential long‐term effects of PAR polymers in IVDD, we treated cultured NPCs with exogenous PAR and performed transcriptome sequencing. KEGG enrichment analysis revealed significant activation of the Hippo signaling pathway, which has been previously implicated in IVDD progression [46, 47]. Subsequent qPCR experiments confirmed the marked suppression of key downstream effectors of the Hippo‐YAP pathway, including *CCN2*, *MYC*, and *CCN1*. These results suggest that, beyond its role in inducing cell death, abnormal PAR accumulation may also contribute to NPC dysfunction and the formation of a degenerative microenvironment through sustained inhibition of pro‐survival signaling pathways critical for cell proliferation and matrix homeostasis [48, 49]. While prior research has largely focused on the cytotoxic effects of PAR accumulation in acute DNA damage responses [50], we propose that it may also play a significant role in chronic degenerative contexts. PAR accumulation could mediate long‐term cellular maladaptation and disrupt tissue homeostasis through mechanisms such as epigenetic modulation or signaling pathway reprogramming. For instance, prolonged PAR exposure may promote cellular senescence, a process closely associated with Hippo pathway activation [51, 52]. Furthermore, given the high sensitivity of the Hippo‐YAP/TAZ pathway to mechanical stress [53, 54], its aberrant activation by PAR might establish a “positive feedback loop” with the mechanical overload microenvironment, potentially exacerbating disc structural deterioration and functional impairment—a hypothesis that awaits further experimental validation. Beyond mechanical stress, other pathological conditions can also induce DNA damage, leading to sustained PAR generation and chronic toxic stress responses. Therefore, further investigation into the biological impact and regulatory mechanisms of PAR accumulation under additional disease contexts is warranted [15]. Additionally, emerging evidence suggests that PAR may contribute to disease progression through mediating protein phase separation [45], representing another promising direction for future research into its pathogenic mechanisms.

Sustained DNA damage and PARP activation typically lead to depletion of intracellular NAD^+^ levels [55]. Consistent with this, our study demonstrated that compression treatment significantly reduced the NAD^+^/NADH ratio in NPCs—an effect that was alleviated by PARP inhibition, suggesting the occurrence of a NAD^+^‐consuming energy crisis due to PARP overactivation during mechanical stress‐induced IVDD. We propose a complex interplay between PAR accumulation and cellular energy dysregulation under mechanical stress: on one hand, persistent PARP activation consumes substantial NAD^+^, while impaired PAR clearance due to PARG degradation further obstructs NAD^+^ regeneration, collectively exacerbating energy stress; on the other hand, NAD^+^ depletion itself weakens the metabolic adaptability required for stress responses. Together, these processes promote NP cell dysfunction and establish a metabolic basis for degeneration under mechanical loading. Notably, the “ROS–DNA damage–PAR” axis and energy dysregulation are both interconnected and independently pathogenic. The ROS scavenger NAC significantly attenuated compression‐induced DNA damage and reversed cell death, indicating that ROS acts as an upstream trigger. In contrast, although supplementation with the NAD^+^ precursor NMN partially alleviated energy stress, it neither effectively reduced DNA damage nor significantly improved cell survival. This suggests that once PAR polymers accumulate beyond a critical threshold, they may directly trigger cytotoxicity and death programs independently of energy deficiency. In other words, NAD^+^ depletion may represent an early metabolic nexus, whereas aberrant PAR accumulation constitutes a key downstream node determining cell fate: even partial restoration of NAD^+^ levels via NMN fails to reverse PAR‐mediated lethal signaling [13]. It is also noteworthy that NAD^+^ is not only a central cofactor in energy metabolism but also an essential substrate for enzymes such as sirtuins, which regulate critical processes including stress resistance, apoptosis, and inflammation [56]. Its decline may directly impair sirtuin activity, further aggravating genomic instability and mitochondrial dysfunction, ultimately forming a vicious cycle with PAR accumulation that synergistically drives IVDD progression [57].

Beyond its role as a key downstream effector of mechanical stress revealed in this study, TRIM25 expression and activity are known to be finely regulated by multiple upstream factors under various pathophysiological conditions. Evidence suggests that its regulatory mechanisms are complex, involving transcriptional, post‐transcriptional, and post‐translational levels. At the transcriptional level, TRIM25 can be directly upregulated by transcription factors such as FOXO4 through promoter binding [58], and may also be upregulated through a positive feedback loop involving the E2F/ATAD2 axis [59]. At the post‐transcriptional level, various non‐coding RNAs can regulate TRIM25 mRNA stability or translation efficiency; for example, lncRNA XIST upregulates TRIM25 expression by sponging miR‐192 [60], while circRNA hsa_circ_0026134 promotes its expression by sequestering miR‐127‐5p [61]. Furthermore, the RNA sensor RIG‐I can also enhance the stability of TRIM25 transcripts [34]. At the post‐translational level, the stability of the TRIM25 protein is dynamically regulated by modifications such as phosphorylation (e.g., MAP3K13‐mediated phosphorylation at Ser12 reduces its polyubiquitination and degradation [62]) and acetylation (e.g., CBP‐mediated acetylation at K392 enhances its interaction with USP7, thereby reducing ubiquitination [63]). In addition, extracellular signals such as IL‐27 [64] and EETs [65] can also induce TRIM25 expression through specific signaling pathways. These studies indicate that TRIM25 expression is likely controlled by a multidimensional regulatory network that integrates multiple upstream inputs, including cytokines, metabolites, non‐coding RNAs, transcription factors, and modifying enzymes. The mechanical compression‐induced upregulation of TRIM25 observed in this study may represent a specific manifestation of this network in the mechanobiological microenvironment, likely involving calcium‐dependent mechanotransduction signals as indicated by our preliminary data. Whether it involves the known regulatory factors or unique pathways still requires further investigation.

Although this study systematically reveals the central role of TRIM25 in mechanical stress‐induced IVDD and its multi‐mechanistic regulatory network, several limitations remain to be addressed in future research. First, the upstream signaling mechanisms through which compression regulates TRIM25 expression have not been fully elucidated. Whether it involves mechanosensitive ion channels (such as Piezo1), integrin–YAP/TAZ signaling, or other mechanosensory molecules requires further validation. Second, beyond Ku80 and PARG, TRIM25 may target additional unidentified substrate proteins involved in IVDD pathogenesis, necessitating systematic screening and identification through untargeted proteomic approaches such as ubiquitinomics. Third, while this study primarily focused on NPCs, whether TRIM25 exerts similar or distinct functions in annulus fibrosus cells or cartilage endplate cells remains unclear. Although in vivo experiments showed that silencing TRIM25 significantly alleviated intervertebral disc degeneration in a rat model, small molecule inhibitors targeting TRIM25 need further development, and their long‐term safety, tissue‐specific delivery strategies, and efficacy in chronic degeneration models that more closely resemble human disease pathology need to be evaluated. Furthermore, the mechanistic validation of the MIF inhibitor 4‐IPP may involve its dual pharmacological effects. 4‐IPP is a covalent inhibitor of both MIF and its structural homolog D‐dopachrome tautomerase (DDT/MIF‐2), and DDT/MIF‐2 also exerts signaling effects through the CD74 receptor [66]. Therefore, the protective effect observed in this study may be partly due to the simultaneous inhibition of DDT, and further studies using specific gene knockout models are needed to further elucidate their respective contributions. Finally, it is important to note that this study used a modified MRI‐based Pfirrmann grading system to assess intervertebral disc degeneration. Although this scoring system has shown good correlation with histological and molecular degeneration markers in our and other studies [41, 67], it is crucial to acknowledge the inherent anatomical and biomechanical differences between human lumbar intervertebral discs and the rat caudal intervertebral disc model. Addressing these issues will facilitate the clinical translation of TRIM25‐targeted therapeutic strategies.

## 4 Materials and Methods

### 4.1 Isolation, Culture of NPCs, and Ethical Approval

Human nucleus pulposus (NP) tissue was obtained from intervertebral disc samples of patients undergoing spinal surgery. Control disc tissues were collected from 40 patients (age range: 17–35 years; 21 females and 19 males) undergoing surgery due to thoracolumbar injury or scoliosis. Degenerated disc tissues were obtained from 48 patients (age range: 32–65 years; 25 females and 23 males) who underwent discectomy for symptoms of nerve compression. The degenerative status of all samples was classified according to the Pfirrmann grading system. The experimental protocol was approved by the Ethics Committee of Tongji Medical College, Huazhong University of Science and Technology, in accordance with the principles of the Declaration of Helsinki. Written informed consent was obtained from all donors.

For primary cell isolation, healthy NP tissues were minced into approximately 1 mm^3^ fragments and digested with 0.2% type II collagenase (Cat. No. 2275MG100, BioFroxx, Germany) at 37°C for approximately 10 h. The cell suspension was then centrifuged at 300 × g for 5 min. The pellet was resuspended and cultured in DMEM/F12 medium supplemented with 10% fetal bovine serum (FBS, Cat. No. 10099–141, Gibco, Invitrogen, New York, USA) and 1% penicillin–streptomycin (Cat. No. C0222, Beyotime, China). Cells were maintained at 37°C in a humidified atmosphere of 5% CO_2_. Upon reaching 70%–80% confluence, the cells were passaged using 0.25% trypsin (Cat. No. 25200114, Gibco, Invitrogen, USA). The isolation and culture procedures were performed in strict accordance with our laboratory's previously established protocols [68, 69]. The purity and phenotypic identity of the isolated NPCs were consistent with our prior characterizations, which verified the expression of specific notochordal markers, including CD24 and KRT18, via immunofluorescence staining [69, 70]. Second‐generation (P2) cells were used for all subsequent experiments to ensure phenotypic stability.

### 4.2 Drugs and Cell Treatment

The following chemical inhibitors were utilized in this study: Z‐VAD (HY‐164388), Chloroquine (CQ, HY‐17589A), NAC (N‐acetylcysteine, HY‐B0215), NMN (Nicotinamide mononucleotide, HY‐F0004), MG‐132 (HY‐13259), and Cycloheximide (HY‐12320) were all purchased from MedChemExpress. Additionally, ABT‐888 (Veliparib, S1004), AG‐014699 (Rucaparib, S4948), 4‐IPP (S2643), and COH34 (E4736) were obtained from Selleck. Purified poly (ADP‐ribose) (PAR) polymer was purchased from Trevigen (Gaithersburg, Maryland, USA; Catalog number: 4336‐100‐01). To verify the cellular internalization of exogenous PAR, a fluorescently labeled PAR probe (PAR‐Cy5.5) was synthesized using an EDC‐mediated coupling method targeting the phosphate backbone. NPCs were incubated with the PAR‐Cy5.5 probe for 24 h, followed by fixation and co‐staining with FITC‐phalloidin (to visualize F‐actin cytoskeleton) and DAPI to confirm intracellular localization.

Cells were subjected to mechanical compression using a custom‐designed compression apparatus equipped with a stainless‐steel pressure chamber, as detailed in our previous publication [27]. Briefly, the system was utilized to apply a static pressure of 1.0 MPa or 1.3 MPa. The magnitude of compressive stress was set at these specific levels based on physiological intradiscal pressure measurements and our previous protocols [27, 71]. Previous in vivo studies have established that intradiscal pressure in the human lumbar spine can rise to approximately 1.1–2.3 MPa during weight‐lifting or flexion [72, 73]. Therefore, 1.0 MPa was selected to simulate a heavy physiological load relevant to occupational stress, while 1.3 MPa represented a severe overload condition to assess dose‐dependent pathological responses. Throughout the compression period, the pressure chamber was continuously infused with a gas mixture consisting of 5% CO_2_ and 95% compressed air to maintain physiological pH and aerobic conditions. Unless otherwise indicated in the figures, cells were exposed to continuous compression for a duration of 36 h prior to subsequent analysis.

### 4.3 Cell Proliferation and Viability Assays

Cell proliferation and viability were assessed using Calcein/PI Cell Viability and Cytotoxicity Assay Kit (C2015M, Beyotime, China) and Cell Counting Kit‐8 (CCK‐8 kit, C0037, Beyotime, China). Cells were seeded in 96‐well or 12‐well plates and treated as required. After treatment, adherent cells were washed with PBS to remove residual media; suspension cells were centrifuged and resuspended in PBS. Cells were then stained with Calcein AM/PI working solution and incubated at 37°C for 30 min in the dark. Fluorescence was observed under a microscope (Calcein AM: Ex/Em = 494/517 nm; PI: Ex/Em = 535/617 nm). For the CCK‐8 assay, 10 µL of reagent was added to each well and incubated for 2 h at 37°C before measuring the absorbance at 450 nm.

### 4.4 RNA Extraction and Real‐Time Quantitative PCR (RT‐qPCR)

Total RNA was isolated from cultured cells or NP tissue samples using the RNApure Total RNA Kit (#RC101, Vazyme, China) according to the manufacturer's instructions. RNA concentration and purity were determined spectrophotometrically using a NanoDrop system. Subsequently, 1 µg of total RNA was reverse‐transcribed into cDNA using the TransScript All‐in‐One First‐Strand cDNA Synthesis SuperMix (#AE311‐02, TransGen, China). Quantitative PCR was performed with the TransStart Tip Green qPCR SuperMix (#AQ601‐01, TransScript, China) on a QuantStudio 5 Real‐Time PCR System (Applied Biosystems, USA). Each reaction was carried out in triplicate under the following cycling conditions: initial denaturation at 95°C for 30 s, followed by 40 cycles of 95°C for 5 s and 60°C for 30 s. Gene expression levels were normalized to β‐actin. The primer sequences used for the amplification of target genes are listed in Table 1.

**TABLE 1:** Primers.

Gene	Primers
Homo CCN2	Forward‐CACCCGGGTTACCAATGACA
	Reverse‐TCCGGGACAGTTGTAATGGC
Homo CCN1	Forward‐CAGGACTGTGAAGATGCGGT
	Reverse‐GCCTGTAGAAGGGAAACGCT
Homo MYC	Forward‐GCAATGCGTTGCTGGGTTAT
	Reverse‐TCCCTCCGTTCTTTTTCCCG
Homo PARG	Forward‐CATGGTATCGCAGCAAACCG
	Reverse‐GAAAGGAGTGACTGGAGCCC
Homo ARH3	Forward‐CCTGCGTCATGTCCAGAGTC
	Reverse‐GTACAAGGCTTCTGTCCGCT
Homo TRIM25	Forward‐CAACTGTGACCACGGCTTTG
	Reverse‐AGCCTTCAGATCCAAGTGGC
Homo TNF	Forward‐GACAAGCCTGTAGCCCATGT
	Reverse‐GGAGGTTGACCTTGGTCTGG
Homo IL1B	Forward‐AACCTCTTCGAGGCACAAGG
	Reverse‐AGATTCGTAGCTGGATGCCG
Homo IL‐6	Forward‐CCTTCGGTCCAGTTGCCTTCT
	Reverse‐TCTGAGGTGCCCATGCTACA
Homo CXCL8/IL‐8	Forward‐GAAGTTTTTGAAGAGGGCTGAGA
	Reverse‐ACCAAGGCACAGTGGAACAA
Homo DDX58/RIG‐I	Forward‐CACCTCAGTTGCTGATGAAGGC
	Reverse‐GTCAGAAGGAAGCACTTGCTACC
Homo GAPDH	Forward‐TTTTGCGTCGCCAGCC
	Reverse‐ATGGAATTTGCCATGGGTGGA
Homo IFNB1	Forward‐AGGACAGGATGAACTTTGAC
	Reverse‐TGATAGACATTAGCCAGGAG
Homo CDKN1A	Forward‐TGTCCGTCAGAACCCATGC
	Reverse‐AAAGTCGAAGTTCCATCGCTC
Homo CDKN2A	Forward‐GATCCAGGTGGGTAGAAGGTC
	Reverse‐CCCCTGCAAACTTCGTCCT

### 4.5 Genetic Knockdown and Knockout Assays

Gene silencing was achieved by transfection with sequence‐specific siRNA or shRNA targeting the gene of interest. Cells were plated in appropriate culture dishes and allowed to adhere for 24 h until they reached 50%–60% confluency before transfection. siRNA duplexes targeting human PARG and ARH3 were purchased from Dharmacon (Lafayette, CO, USA). sgRNA targeting TRIM25 was purchased from Gibco (Shanghai, China). Transfections were performed using Lipofectamine 2000 transfection reagent (Invitrogen, Carlsbad, CA, USA) according to the manufacturer's instructions. Briefly, an appropriate amount of siRNA or shRNA was diluted in Opti‐MEM I Reduced Serum Medium (Gibco, USA) and gently mixed. An equal volume of diluted Lipofectamine 2000 was then mixed with the siRNA or shRNA solution, incubated at room temperature for 20 min, and the complex was added dropwise to the cells. Six to eight hours after transfection, the medium was replaced with fresh complete growth medium. Knockdown efficiency was routinely verified at the mRNA and protein levels 48–72 h after transfection by quantitative real‐time PCR (qRT‐PCR) and Western blot analysis, respectively. A complete list of all RNAi sequences used in this study, unless otherwise stated, is listed in Table 2.

**TABLE 2:** RNAi Sequences.

RNAi Sequence name	target‐sequence (sense of 5'‐3')
Homo‐shRNA‐PARG	TACCAGGGTT ACTGTTTGAGG
Homo‐shRNA‐TRIM25#1	GCTTTCGAGACGATGATTATT
Homo‐shRNA‐TRIM25#2	AGGATGAGGTCGGGTACATAT
Homo‐siRNA‐TRIM25	AUGGAUUUUCUCUAAGAGGAA
Homo‐sgRNA‐TRIM25	GGGAGCCACCCGCCGACGTC
Homo‐siRNA‐DDX58/RIG‐I	CCGGCACAGAAGUGUAUAUTT
Rat‐shRNA‐TRIM25	GGTGGAGCAGCTACAACAA

### 4.6 RNA Sequencing (RNA‐seq)

Total RNA was isolated from tissue samples using TRIzol reagent (#T9424, Sigma–Aldrich, USA). RNA purity was assessed with a Nanodrop ND‐2000 spectrophotometer (#ND‐2000, Thermo Scientific, USA) based on A260/A280 ratios. RNA integrity was evaluated using an Agilent 4150 Bioanalyzer system (#G2992AA, Agilent Technologies, USA), which provides RNA Integrity Number (RIN) values. A paired‐end RNA‐seq library was constructed with the ABclonal mRNA‐seq Lib Prep Kit (#RK20350, ABclonal, China). Briefly, mRNA was enriched from 1 µg of total RNA using oligo(dT) magnetic beads. The purified mRNA was then fragmented in fragmentation buffer under elevated temperature. First‐strand cDNA was synthesized from the fragmented mRNA using random hexamer primers and reverse transcriptase. Second‐strand cDNA synthesis was subsequently performed using DNA polymerase I, RNase H, and dNTPs. The double‐stranded cDNA fragments were end‐repaired, A‐tailed, and ligated to Illumina‐compatible adapters. The adapter‐ligated fragments were then amplified by PCR. The amplified library was purified, and its quality and size distribution were verified using the Agilent 4150 Bioanalyzer. Sequencing was performed on an Illumina NovaSeq 6000 with a paired‐end 150 bp (PE150) read configuration. Data generated from the sequencing platforms were subjected to subsequent bioinformatic analysis.

### 4.7 Western Blot

Protein extracts from cultured cells or tissue samples were prepared using RIPA lysis buffer (Cat# P0013B, Beyotime, China,) supplemented with 1:100 dilution of protease inhibitor cocktail (Cat# HY‐K0010, MedChemExpress, China). The lysates were incubated on ice for 30 min and subsequently sonicated to ensure complete disruption. Following centrifugation at 12 000 × g for 10 min at 4°C, the supernatant was collected and mixed with 5× SDS loading buffer (Cat# LT101, Epizyme, China). The samples were denatured by boiling at 99°C for 10 min and stored at −80°C until use. Proteins were separated by SDS‐PAGE using pre‐cast gels and then transferred onto nitrocellulose (NC) membranes (Millipore, USA) using a semi‐dry transfer system (Cat# 10112001, WIX, China). The membranes were blocked with rapid blocking buffer (Cat# PS108, Epizyme, China) for 15 min at room temperature and then incubated with primary antibodies overnight at 4°C. Unless otherwise stated, detailed information regarding all antibodies used is provided in Table 3. After washing, the membranes were probed with corresponding horseradish peroxidase (HRP)‐conjugated secondary antibodies. Protein bands were visualized using an enhanced chemiluminescence (ECL) substrate (Cat# P0018FS, Beyotime, China) and imaged with a chemiluminescence detection system (ChemiDoc MP, Bio‐Rad, USA). Quantitative analysis of the bands was performed using Image Lab software.

**TABLE 3:** Antibodies.

Product name	Catalog	Manufacturer	Dilution Ratio
γ‐H2AX	ab81299	abcam	1:1000 (WB); 1:200 (IF)
PAR	4335‐MC‐100	Trevigen	1:1000 (WB); 1:200 (IF)
AIF	17984‐1‐AP	proteintech	1:2000 (WB); 1:200 (IF)
MIF	20415‐1‐AP	proteintech	1:2000 (WB); 1:200 (IF)
Histone H3	R381432	ZENBIO	1:2000
PARP1	R380451	ZENBIO	1:2000
PARG	27808‐1‐AP	proteintech	1:2000 (WB); 1:200 (IF)
ARH3	ab224751	abcam	1:1000
Tubulin	M20005	abmart	1:10000
Ub	10201‐2‐AP	proteintech	1:2000
TRIM25	R25984	ZENBIO	1:2000 (WB); 1:200 (IF)
KU80	R381198	ZENBIO	1:2000 (WB); 1:100 (IF)
HA	51064‐2‐AP	proteintech	1:3000
FLAG	66008‐4‐Ig	proteintech	1:2000
RIG‐I	84861‐5‐RR	proteintech	1:2000 (WB); 1:200 (IF)
IL1B	516288	ZENBIO	1:1000 (WB); 1:100 (IF)
TNF	346654	ZENBIO	1:1000
IL‐6	500286	ZENBIO	1:2000
IL‐8	ab289967	abcam	1:2000
YAP	13584‐1‐AP	proteintech	1:2000
p‐YAP	80694‐2‐RR	proteintech	1:2000
ATM	27156‐1‐AP	proteintech	1:2000
p‐ATM	Ab315019	abcam	1:2000
ATR	19787‐1‐AP	proteintech	1:2000
p‐ATR	86330‐1‐RR	proteintech	1:2000
p‐CHK1	28805‐1‐AP	proteintech	1:2000
p‐CHK2	81740‐1‐RR	proteintech	1:2000
Aggrecan	13880‐1‐AP	proteintech	1:100 (IF)
Collagen‐2	28459‐1‐AP	proteintech	1:100 (IF)
Cleaved caspase‐3	87055‐4‐RR	proteintech	1:2000 (WB)
p‐MLKL	R382136	ZENBIO	1:2000 (WB)
GSDMD‐N	R40133	ZENBIO	1:2000 (WB)

### 4.8 Co‐Immunoprecipitation (Co‐IP) and Mass Spectrometry Analysis

For co‐immunoprecipitation assays, cells were lysed using IP‐specific lysis buffer (#P0013, Beyotime, China) supplemented with protease and phosphatase inhibitors (#P1049, Beyotime, China) and PMSF (#ST506, Beyotime, China). Cell lysates were incubated with magnetic beads conjugated with protein A/G (#HY‐K0202, MedChemExpress, USA) and specific antibodies targeting the protein of interest. After extensive washing with lysis buffer, the immunocomplexes were eluted and separated by SDS‐PAGE, followed by transfer onto PVDF membranes. The membranes were blocked for 1 h at room temperature and then incubated overnight at 4°C with specific primary antibodies. After washing with TBST (Tris‐buffered saline containing 0.1% Tween 20), the membranes were incubated with HRP‐conjugated secondary antibodies for 1 h at room temperature. Protein bands were visualized using enhanced chemiluminescence reagent (#HY‐K1005, MedChemExpress, USA) on a ChemiDoc MP Imaging System (Bio‐Rad, USA), and band intensity was quantified using ImageJ software.

To identify proteins that interact with PARG, co‐immunoprecipitation was performed in NPCs using antibodies specifically targeting PARG bound to protein A/G magnetic beads. The beads were washed three times with ice‐cold PBS and then subjected to on‐bead digestion. Proteins were denatured, reduced, and alkylated in a reaction buffer containing 1% SDC, 100 mmol/L Tris‐HCl (pH 8.5), 10 mmol/L TCEP, and 40 mmol/L CAA at 95°C for 10 min. Tryptic digestion was carried out overnight at 37°C. The resulting peptides were purified using homemade SDB desalting columns. LC‐MS/MS analysis was performed on an Easy‐nLC 1200 system coupled to a Q Exactive HF‐X mass spectrometer (Thermo Scientific). Raw data were processed using MaxQuant software (version 1.6.6) and searched against the Andromeda database. The “proteingroups.txt” output from MaxQuant was used for downstream analysis. Proteins exhibiting expression changes beyond the threshold of mean ± 1.64 × SD were considered significantly altered.

### 4.9 Immunofluorescence Staining

Cultured cells or paraffin tissue sections were fixed with 4% paraformaldehyde for 10 min at room temperature, permeabilized with 0.3% Triton X‐100 for 10 min, and blocked with 5% bovine serum albumin for 30 min. The samples were then incubated with primary antibodies for 2 h at room temperature or overnight at 4°C. Antibody dilutions used are detailed in Table 3. After incubation, samples were washed three times with PBST and incubated with appropriate fluorophore‐conjugated secondary antibodies for 1 h at room temperature, protected from light. Following three washes with PBS, nuclei were counterstained with DAPI (Cat# C1002, Beyotime, China) for 5 min. Images were acquired using a laser scanning confocal microscope (Olympus, Japan). Quantitative analysis of mean fluorescence intensity was performed using ImageJ software, with data collected from at least three independent visual fields per condition.

### 4.10 Comet Assay and DNA Damage Evaluation

DNA damage was evaluated using the single‐cell gel electrophoresis (comet assay) technique with a commercial kit (Comet Assay Kit, C2041S, Beyotime Biotechnology, China). The assay was performed under dim light to prevent additional DNA damage. Briefly, harvested cells were centrifuged to obtain a cell pellet, which was then resuspended in PBS. The first layer of the comet slide was prepared with 1% normal melting point agarose. Subsequently, 70 µL of low melting point agarose containing approximately 1 × 10^4^ cells was evenly spread over the first layer using a pipette tip and immediately covered with a coverslip. The slides were maintained at 4°C in the dark for 10 min to solidify. After solidification, the coverslips were carefully removed, and the slides were immersed in pre‐chilled lysis buffer (provided in the kit) at 4°C for 2 h. Following lysis, the slides were placed in an alkaline unwinding solution for 40 min at 4°C to allow DNA denaturation. Electrophoresis was conducted in the same alkaline buffer at 25 V for 30 min. Afterward, the slides were neutralized and then stained with Propidium Iodide Solution for 15 min. The comets were visualized using a fluorescence microscope (Nikon, Japan). Quantification of DNA damage was performed using ImageJ software. Parameters including Tail DNA (%) and Tail Moment were calculated from three independent experiments.

In parallel, oxidative DNA damage was quantified by measuring 8‐OHdG levels using a competitive enzyme‐linked immunosorbent assay (ELISA) kit (ab285254, Abcam, UK). Genomic DNA was isolated from cell pellets, enzymatically hydrolyzed to nucleosides, and diluted according to the manufacturer's instructions. The assay was performed on the hydrolyzed samples in duplicate. Absorbance was measured at 450 nm, and 8‐OHdG concentrations were determined by interpolation from a standard curve. Data are presented as the ratio of 8‐OHdG to deoxyguanosine (dG) or as 8‐OHdG per µg of DNA, from three independent experiments.

### 4.11 Flow Cytometry Detection of ROS

Cellular reactive oxygen species (ROS) levels were assessed using a Reactive Oxygen Species Detection Kit (S0034S, Beyotime, China). Treated cells were incubated with 10 µm DCFH‐DA (diluted 1:1000 in probe dilution buffer) at 37°C for 20 min. After incubation, cells were thoroughly washed to remove extracellular probe. A ROS positive control (Rosup) was applied only to the designated samples for 20–30 min. Finally, cells were analyzed by flow cytometry using 488 nm excitation and 525 nm emission detection (FITC channel).

### 4.12 NAD^+^/NADH Assessment

The NAD^+^/NADH ratio was assessed in NPCs using the NAD^+^/NADH Assay Kit (S0175, Beyotime, China). Briefly, approximately 1 × 10^6^ cells were lysed with 200 µL of ice‐cold extraction buffer. The lysate was centrifuged at 12 000 × g for 10 min at 4°C, and the supernatant was collected for analysis. A standard curve was prepared using serial dilutions of NADH. For total NAD^+^/NADH measurement, 20 µL of sample was added into a 96‐well plate, mixed with 90 µL of alcohol dehydrogenase working solution, and incubated at 37°C for 10 min protected from light. Then, 10 µL of chromogenic solution was added to each well and incubated for another 10–20 min at 37°C. To quantify NADH alone, an aliquot of the supernatant was heated at 60°C for 30 min to decompose NAD^+^ before the same reaction procedure. Absorbance was measured at 450 nm, and concentrations were calculated based on the standard curve. The NAD^+^/NADH ratio was derived from the values of total NAD^+^ + NADH and NADH alone.

### 4.13 Animal Models and in vivo Interventions

An established rat model of intervertebral disc degeneration was utilized as previously described [74]. Briefly, Sprague–Dawley rats (weighing 250±20 g) were randomly assigned to the experimental groups (Sham, Compression, and Treatment groups) using a standard random number table method. The rats were anesthetized, and carbon fiber rings were affixed to the coccygeal vertebrae (Co7 and Co9) using sterile 0.8 mm Kirschner wires. Axial loading was applied via four calibrated springs (0.50 N/mm) mounted on each rod. The applied stress was set to 1.3 MPa, and spring deflection was calculated based on the standard mechanical equation referenced in the original protocol. Sham‐operated animals underwent the identical surgical procedures, including the implantation of Kirschner wires and the attachment of the external compression device. However, the springs on the device were left uncompressed to maintain a zero‐load condition, thereby strictly controlling for the effects of surgical trauma and the physical presence of the apparatus. Lentiviral vectors carrying shTRIM25 or oePARG (1 × 10^8^ TU/mL; GeneChem, China) were administered weekly via intradiscal injection over a period of four weeks. A total volume of 2 µL viral suspension was delivered vertically into the intervertebral disc using a 33‐gauge needle at an approximate depth of 5 mm. Magnetic resonance imaging was performed at week 8 post‐intervention, and tissue samples were harvested at the endpoint for histological and immunofluorescence analyses. To minimize observer bias, all outcome assessments—including MRI grading and histological scoring—were performed by two independent investigators who were blinded to the specific group assignments. Following surgery, all animals were monitored daily and exhibited normal recovery patterns, maintaining regular food intake and grooming behaviors. The external compression device was designed to minimize physical interference, ensuring that all animals were permitted free cage activity without restriction on weight‐bearing or significant impairment of daily mobility. All experimental procedures were approved by the Institutional Animal Care and Use Committee and conducted in accordance with relevant ethical guidelines and regulations.

### 4.14 Evaluation of IVDD

At week 8 post‐surgery, all rats underwent magnetic resonance imaging (MRI) for morphological and structural evaluation of intervertebral discs. During imaging, animals were anesthetized and placed in a prone position with the tail extended. Disc degeneration was assessed based on the Pfirrmann grading system, and the disc height index (DHI) was calculated to quantify changes in disc morphology. Following MRI, euthanasia was humanely performed via an overdose of 3% pentobarbital sodium. The caudal spine segments were carefully dissected and collected for subsequent histological processing. The harvested tissues were fixed in 4% paraformaldehyde, decalcified in EDTA solution, dehydrated through a graded ethanol series, and embedded in paraffin. Sections were cut at a thickness of 4 µm using a microtome and subjected to hematoxylin and eosin (H&E) staining, Safranin O–Fast Green staining, and Masson's trichrome staining. These staining protocols enabled detailed evaluation of structural integrity, proteoglycan content, and collagen distribution within the disc tissues. Histological scoring of disc degeneration was performed in a blinded manner according to established criteria as previously described [75].

### 4.15 Statistical Analysis

All data are presented as the mean ± standard error of the mean (SEM) from at least three independent experiments. Statistical significance between groups was determined using a two‐tailed Student's *t*‐test, one‐way analysis of variance (ANOVA), or two‐way ANOVA. All statistical analyses and graph generation were performed using GraphPad Prism version 9. Significance levels are denoted as follows: ^*^ *p* < 0.05, ^**^ *p* < 0.01, ^***^ *p* < 0.001; “ns” indicates not significant.

## Author Contributions

Conceptualization was carried out by Z.C. and Y.Z. Methodology was developed by Z.C., P.S., and K.Z. Investigation was performed by W.B.W., X.C., and Z.Y. Visualization was conducted by Z.C., H.G., and W.W. Supervision was provided by Y.Z. and C.Y. The original draft was written by Z.C. and W.B.W., and review and editing were carried out by Z.C., H.G., and Y.Z.

## Conflicts of Interest

The authors declare no conflicts of interest.

## Supporting information




**Supporting File**: advs74502‐sup‐0001‐SuppMat.docx.

## Data Availability

The data that support the findings of this study are available from the corresponding author upon reasonable request.
