# Supplementary material for: Elevated TRIM25 Impairs Poly (ADP‐ribose) Metabolism via PARG Degradation and Mediates Compression‐Induced Intervertebral Disc Degeneration
Source: Adv Sci (Weinh). 2026 Feb 17;13(25):e19248. doi: 10.1002/advs.202519248 (PMC13137795; doi:10.1002/advs.202519248)
Supplement: Supplementary file 1 — Supporting File: advs74502‐sup‐0001‐SuppMat.docx. [file ADVS-13-e19248-s001.docx]

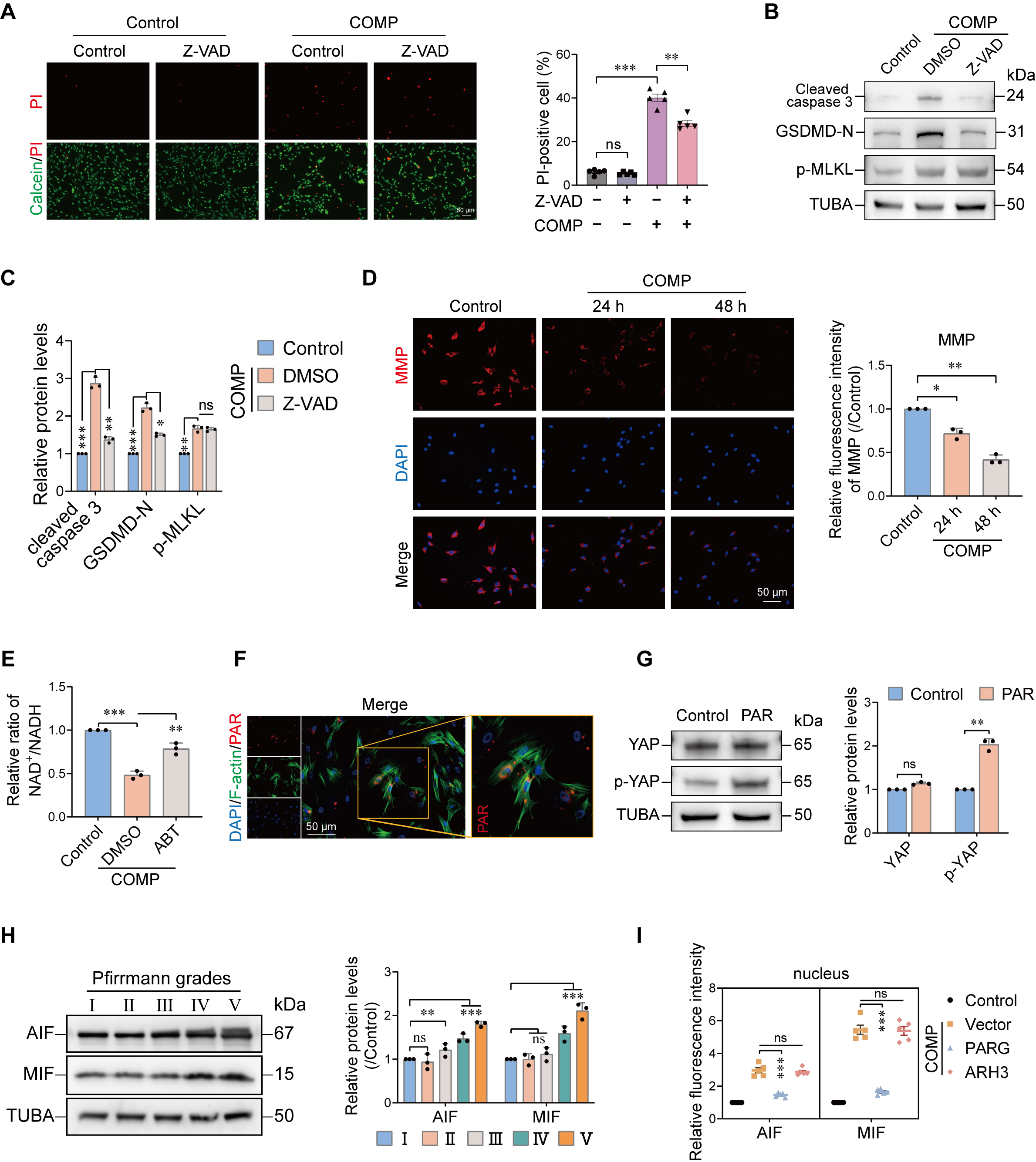


**Figure S1.** (**A**) Calcein-AM/PI staining showing the effect of the pan-caspase inhibitor Z-VAD (20 μM) on compression-induced cell death. Scale bar: 50 μm. n=5. (**B-C**) Western blot analysis (**B**) and quantitative analysis (**C**) of cleaved caspase 3, GSDMD-N, and p-MLKL levels under mechanical stress with or without Z-VAD treatment. n=3. (**D**) Mitochondrial membrane potential (MMP) levels in NPCs under mechanical stress were detected using Mito-Tracker Red CMXRos after 24 and 48 hours. Scale bar: 50 μm. n=3. (**E**) Effect of ABT-888 on the NAD⁺/NADH ratio in NPCs under mechanical compression. n=3. (**F**) Representative immunofluorescence images showing the uptake of fluorescently labeled PAR. Human NPCs were incubated with synthesized PAR-Cy5.5 (red) for 24 hours, followed by co-staining with FITC-phalloidin (F-actin, green) and DAPI (nuclei, blue). Merged images show that PAR-Cy5.5 is localized within the cytoplasm. Scale bar: 50 μm. (**G**) Western blot analysis showing the protein expression levels of phosphorylated YAP (p-YAP) and total YAP in NPCs treated with exogenous PAR compared to the control group. The quantitative analysis (right panel) displays the ratio of p-YAP to Total YAP. n=3. (**H**) Western blot analysis shows a progressive increase in AIF and MIF protein expression in human degenerated intervertebral disc tissues with advancing degeneration grade, indicating a positive correlation with the severity of disc degeneration. n=3. (**I**) Quantitative analysis of the relative nuclear fluorescence intensity of AIF and MIF in compressed NPCs, showing that PARG overexpression, but not ARH3, inhibited the nuclear accumulation of AIF and MIF. n=5. All values are presented as mean ± SEM. *p < 0.05, **p < 0.01, ***p < 0.001, and ns means not significant.


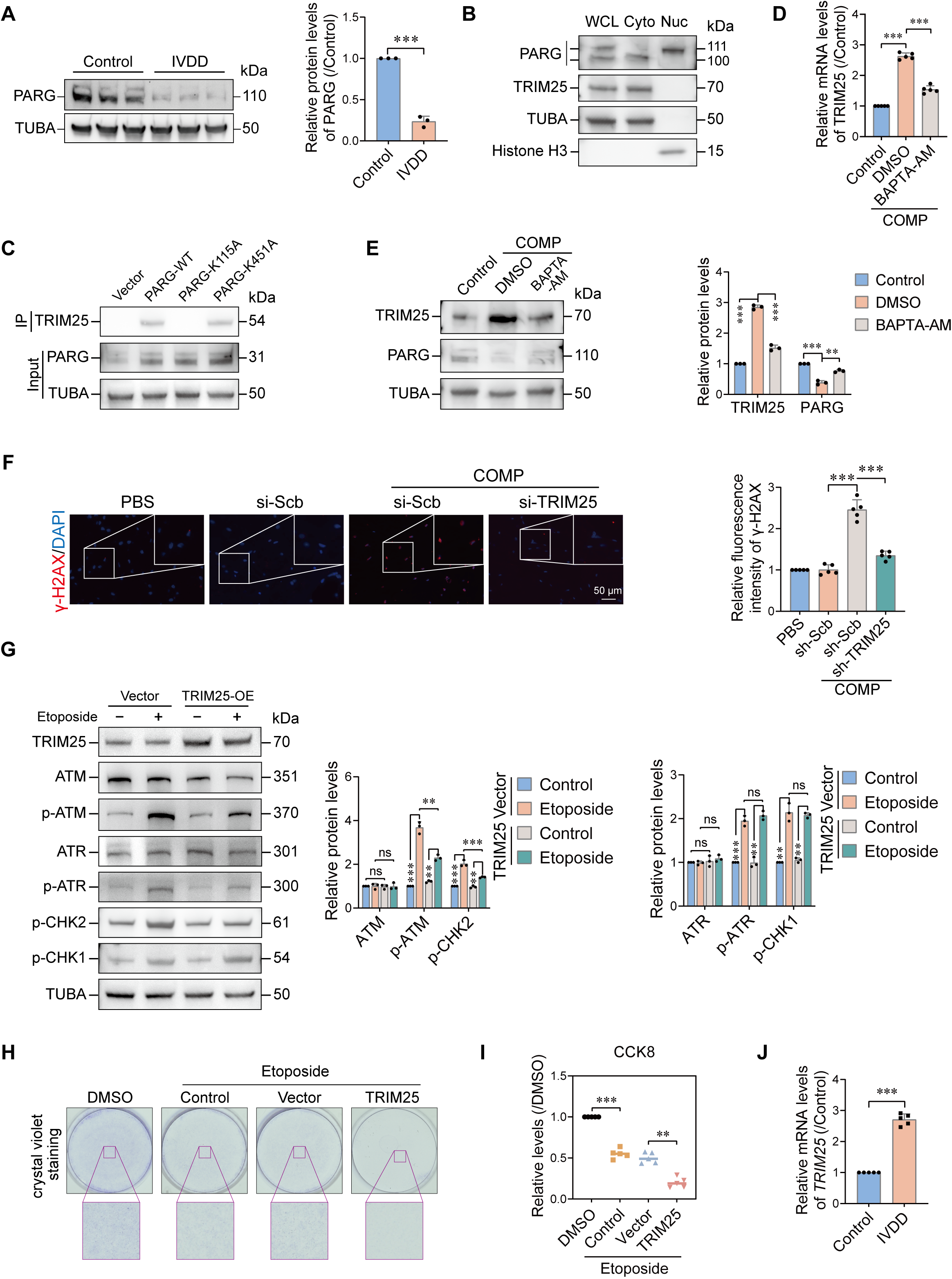


**Figure S2.** (**A**) Western blot demonstrates significantly reduced PARG protein expression in human degenerated disc tissues compared to healthy controls. n=3. (**B**) Nuclear-cytoplasmic fractionation followed by Western blot analysis in NPCs. Histone H3 (nuclear marker) and TUBA (cytoplasmic marker) controls confirm effective separation. The results demonstrate that the full-length PARG isoform (~111 kDa) is predominantly enriched in the nuclear fraction (Nuc), whereas shorter PARG isoforms (~100 kDa) and TRIM25 are localized in the cytoplasmic fraction (Cyto). (**C**) Co-immunoprecipitation (Co-IP) assays showing that TRIM25 maintained a strong interaction with both wild-type PARG and the K451A mutant, but the K115A mutation completely abolished this interaction. (**D-E**) NPCs were pretreated with the intracellular calcium chelator BAPTA-AM prior to mechanical compression. RT-qPCR (**D**) and Western blot (**E**) analyses reveal that blocking calcium signaling effectively abolishes the compression-induced upregulation of TRIM25 at both mRNA and protein levels, identifying calcium influx as a critical upstream trigger. n=3. (**F**) Representative immunofluorescence images and quantitative analysis showing that TRIM25 knockdown alleviates compression-induced increase of γ-H2AX. Scale bar: 50 μm. n=5. (**G**) Western blot analysis of key DNA damage response kinases in NPCs treated with the DSB inducer Etoposide (20 μM, 2 h). TRIM25 overexpression selectively suppressed the phosphorylation of ATM and its downstream target CHK2, while the activation of the ATR-CHK1 axis remained largely unaffected. n=3. (**H**) Representative crystal violet staining images evaluating cell survival following acute DSB induction by Etoposide. (**I**) Cell viability was assessed using the CCK-8 assay in TRIM25-overexpressing and vector control cells under Etoposide treatment. n=5. (**J**) RT-qPCR showed the mRNA levels of TRIM25 in healthy or degenerated human intervertebral disc tissue. n=5. All data are presented as mean ± SEM. *p < 0.05, **p < 0.01, ***p < 0.001, and ns means not significant.


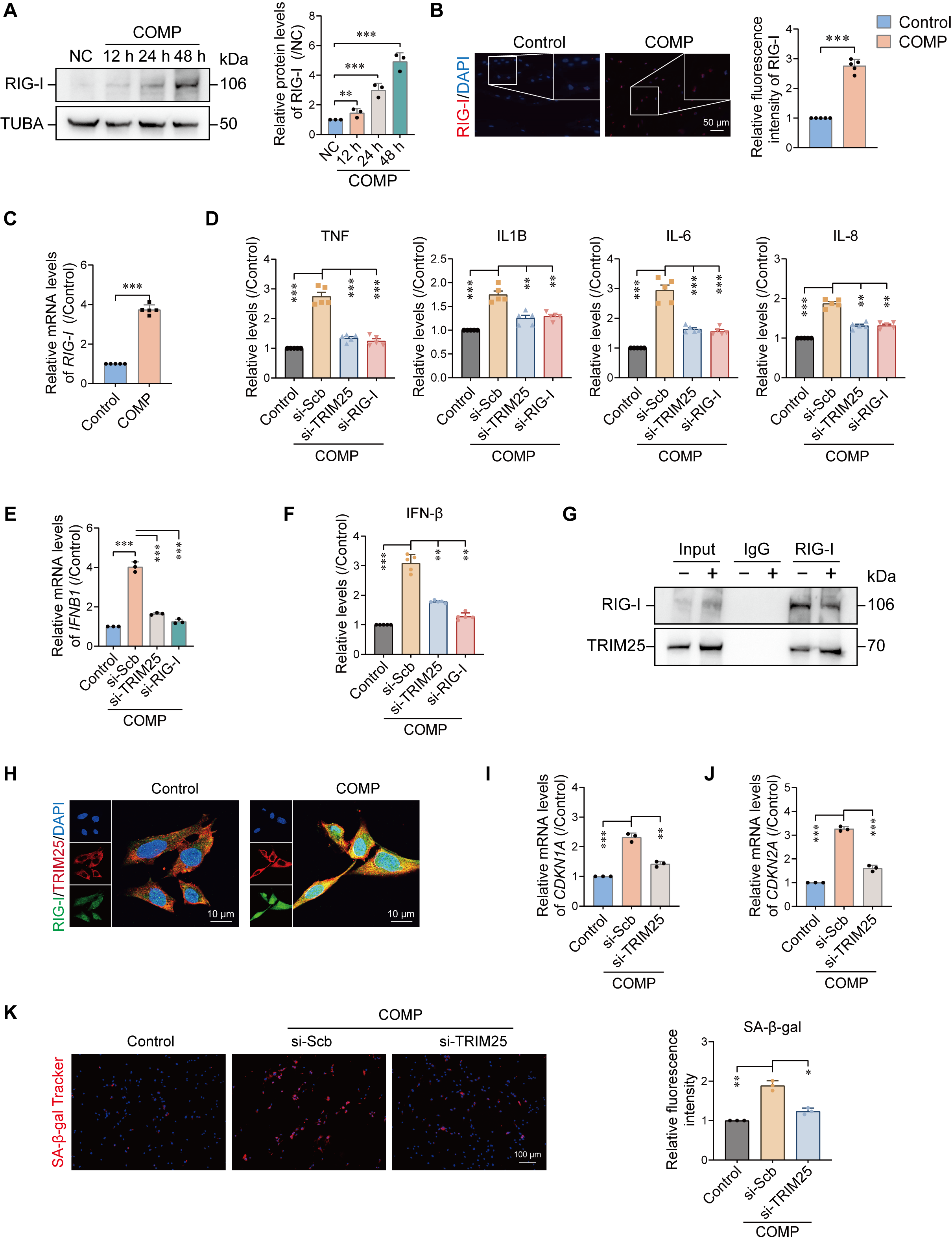


**Figure S3.** (**A**) Western blot indicates a time-dependent upregulation of RIG-I protein expression in NPCs following 12, 24, and 48 hours of mechanical compression. n=3. (**B**) Representative immunofluorescence images and quantitative analysis confirming the upregulation of RIG-I expression in NPCs induced by mechanical compression. Scale bar: 50 μm. n=5. (**C**) RT-qPCR analysis confirms that mechanical compression upregulates RIG-I mRNA expression in NPCs. n=5. (**D**) Enzyme-linked immunosorbent assay (ELISA) analysis of TNF, IL1B, IL-6, and IL-8 levels in the cell culture supernatants of NPCs. Cells were subjected to mechanical compression following transfection with si-TRIM25 or si-RIG-I. The results indicate that mechanical compression significantly promotes the extracellular release of these proinflammatory cytokines, whereas knockdown of TRIM25 or RIG-I effectively attenuates this secretory phenotype. n=5. (**E**) RT-qPCR analysis of *IFNB1* transcript levels. The compression-induced upregulation of *IFNB1* was effectively suppressed by transfection with si-TRIM25 or si-RIG-I. n=5. (**F**) ELISA quantification showing that TRIM25 or RIG-I silencing attenuates compression-induced IFN-β secretion in NPCs. n=5. (**G**) Co-immunoprecipitation assays demonstrate an enhanced interaction between TRIM25 and RIG-I upon mechanical compression. (**H**) Immunofluorescence analysis indicates that mechanical compression promotes the co-localization of TRIM25 and RIG-I within NPCs. Scale bar: 10 μm. (**I-J**) RT-qPCR analysis of key senescence markers, *CDKN2A* and *CDKN1A*. The upregulation of these markers induced by mechanical stress was effectively reversed by TRIM25 silencing, confirming that TRIM25 contributes to compression-induced premature senescence. n=3. (**K**) Representative images of Senescence-Associated β-galactosidase (SA-β-gal) staining. Mechanical compression significantly increased SA-β-gal activity (indicated by SA-β-gal fluorescent tracker), whereas TRIM25 knockdown effectively attenuated this senescence phenotype. Scale bar: 100 μm. n=3. All data are presented as mean ± SEM. *p < 0.05, **p < 0.01, ***p < 0.001, and ns means not significant.


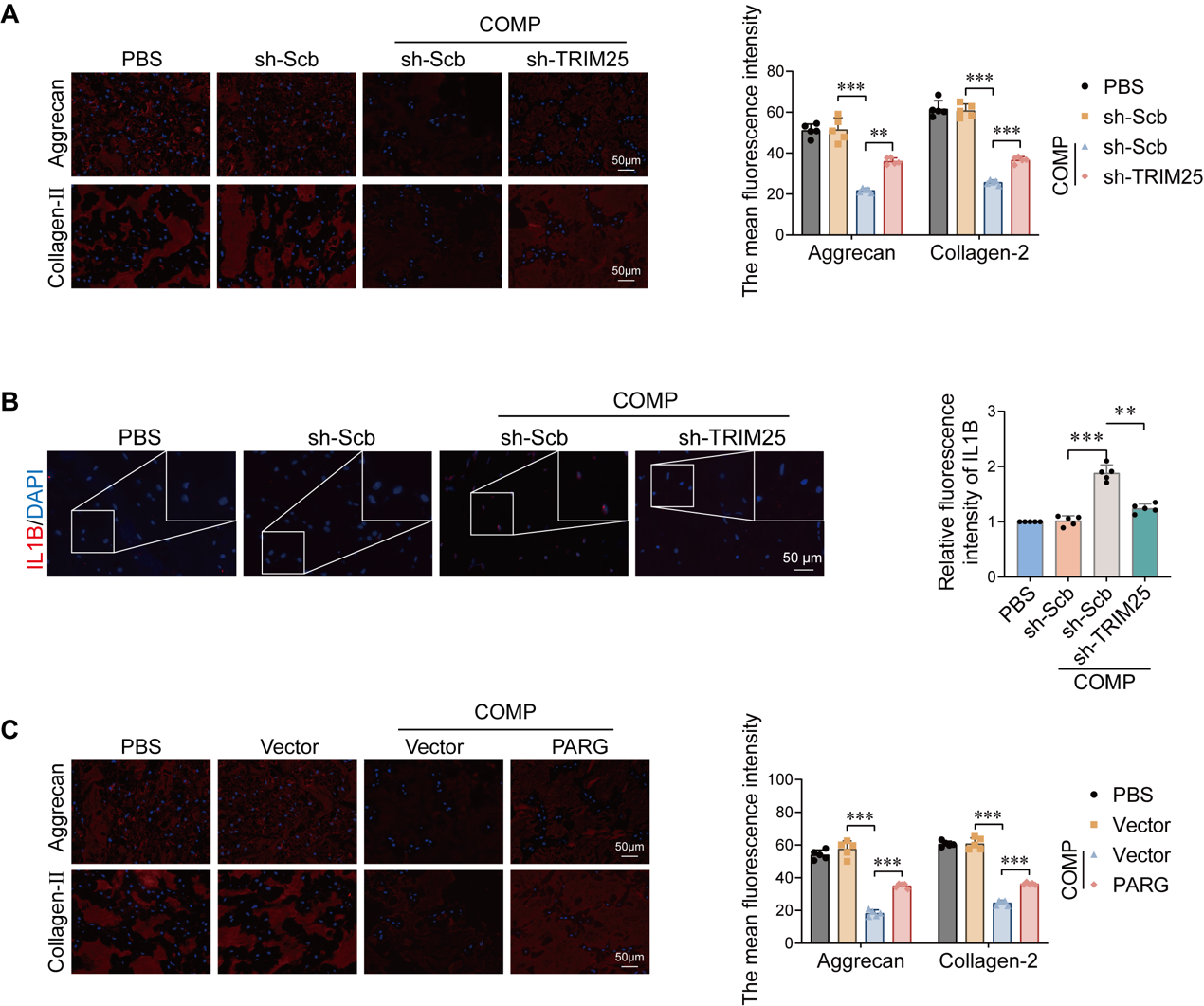


**Figure S4.** (**A**) Representative immunofluorescence images and quantitative analysis of Type II collagen (Collagen-II) and Aggrecan. Mechanical compression reduced fluorescence intensity compared to controls, whereas TRIM25 knockdown restored the expression levels of these matrix components. Scale bar: 50 μm. n=5. (**B**) Immunofluorescence staining shows that TRIM25 knockdown attenuated the upregulation of IL1B expression in rat discs induced by compression. Scale bar: 50 μm. n=5. (**C**) Immunofluorescence analysis of extracellular matrix proteins. The compression-induced decrease in Collagen-II and Aggrecan signals was prevented in the PARG overexpression group. Scale bar: 50 μm. n=5.All data are presented as mean ± SEM. **p < 0.01, ***p < 0.001.


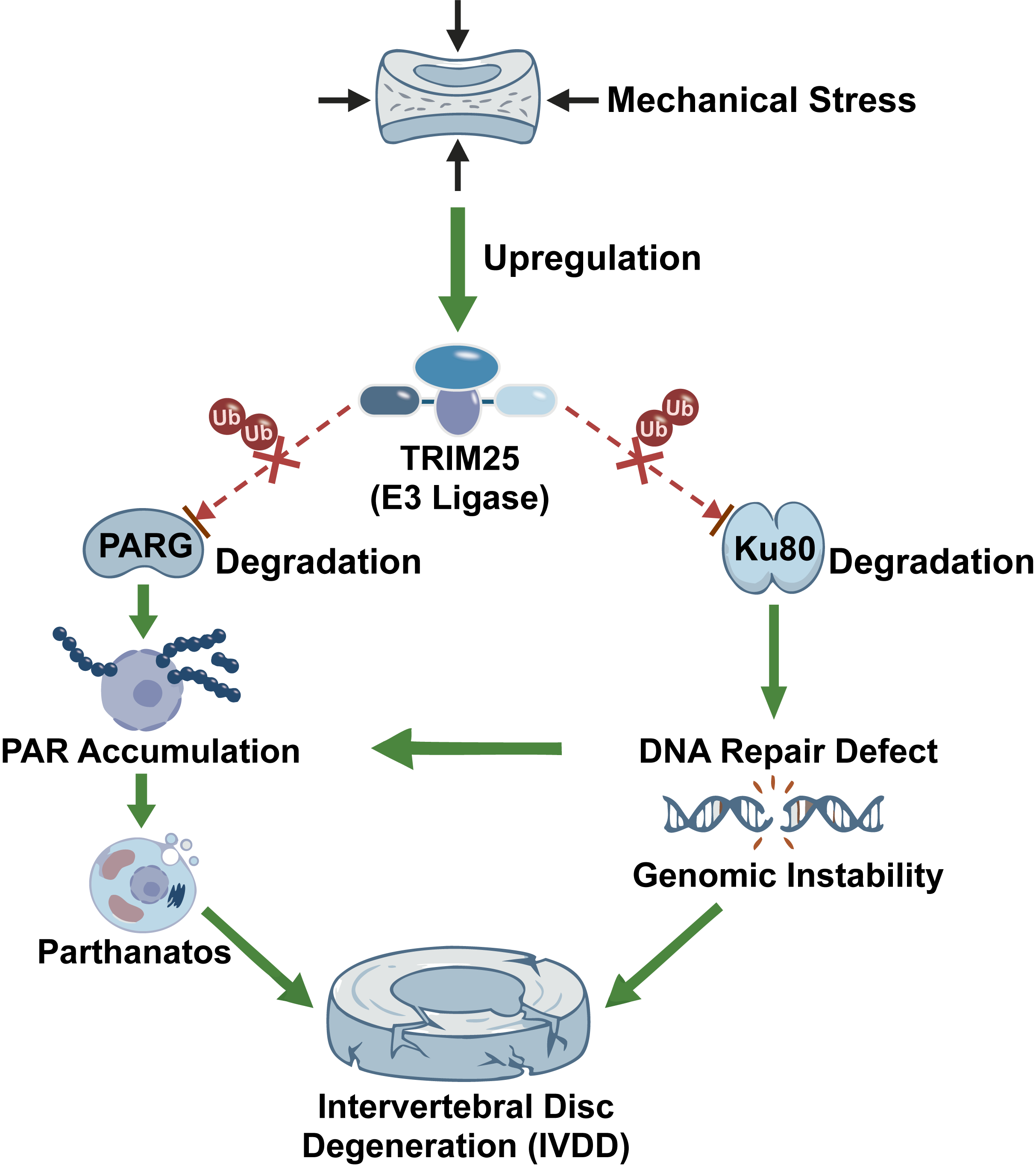


**Figure S5.** TRIM25 acts as a multifunctional hub driving intervertebral disc degeneration under mechanical stress. Mechanical compression significantly upregulates TRIM25 expression, establishing it as a key E3 ubiquitin ligase platform. TRIM25 targets PARG and Ku80 via distinct molecular interfaces, triggering their ubiquitination and degradation. This process synergistically promotes genomic instability and dysregulates PAR metabolism, thereby accelerating parthanatos in NPCs and the progression of IVDD.
